# Person-directed burnout intervention for nurses: A systematic review of psychoeducational approaches

**DOI:** 10.1371/journal.pone.0322282

**Published:** 2025-05-09

**Authors:** Ili Binti Abdullah Sharin, Norehan Binti Jinah, Pangie Anak Bakit, Izzuan Khirman Bin Adnan, Nor Haniza Binti Zakaria, Siti Zubaidah Binti Ahmad Subki, Nursyahda Binti Zakaria, Kun Yun Lee

**Affiliations:** Centre of Leadership and Professional Development, Institute for Health Management, National Institutes of Health, Selangor, Malaysia; University of Hafr Al-Batin, SAUDI ARABIA

## Abstract

**Background:**

Nurse burnout is a pervasive issue impacting their well-being and patient care quality. Recognized by the World Health Organization as an “occupational phenomenon,” burnout results from inadequately managed chronic workplace stress and manifests as emotional exhaustion, depersonalization, and reduced personal accomplishment. This can lower the quality of life and increase turnover. Effective interventions are vital to overcome nurse burnout and its consequences.

**Objective:**

This systematic review explored and analyzed the effectiveness of person-directed psychoeducational interventions in reducing nurse burnout.

**Materials and methods:**

A comprehensive search of five databases was conducted for studies published between 2014 and 2023, following PRISMA guidelines. Eligible studies that reported outcomes of psychoeducational interventions using validated evaluation tools were included. Data were extracted using standardized forms, and quality was assessed with Joanna Briggs Institute critical appraisal tools. A thematic narrative synthesis was performed.

**Results:**

27 studies met the inclusion criteria. Interventions including mindfulness-based interventions and cognitive-behavioral therapy, delivered either in combination or on their own, were shown to be effective in reducing nurses’ burnout in 24 studies. However, the sustainability of these effects varied, with limited long-term follow-up data. Additionally, delivery formats (physical, digital, or combined), also influenced effectiveness, suggesting the importance of tailored interventions to specific contexts and needs of the target population.

**Conclusions:**

Psychoeducational interventions effectively reduce nurse burnout but need further investigation to ensure long-term sustainability. Future research should target diverse settings, incorporate objective and subjective outcome measures, and explore a broader range of interventions to strengthen evidence of burnout management strategies.

## Introduction

### Background

Burnout among nurses carries significant implications [1]. Recognized by the World Health Organization (WHO) as an “occupational phenomenon,” nurse burnout results from chronic workplace stress that is inadequately managed [2]. In the International Classification of Diseases, 11th Revision (ICD-11), burnout is defined as feelings of exhaustion, depersonalization, and diminished personal accomplishment [3]. According to Maslach’s conceptualization [4], emotional exhaustion (EE) entails feeling emotionally drained and lacking in emotional reserves, while depersonalization (DP) or cynicism involves a negative and detached response to others. Reduced personal accomplishment (PA) refers to declined feelings of competence and performance at work. The consequences of nurse burnout are far-reaching; it lowers quality of life, performance levels, and organizational commitment. Worse still, it may escalate their intention to leave [5], potentially exacerbating staff turnover and the quality of nursing care [6].

In 2020, the global pooled prevalence of nurse burnout was 11.2% [7]. However, preceding studies evaluating burnout symptoms indicated rates as high as 40.0% [8,9]. Post-COVID-19 pandemic, the rate has soared to as high as 68.0% [10]. Its prevalence in Asia is particularly worrying due to the challenging working conditions in the region, including low nurse-patient ratios and an aging population. This viewpoint is supported by a meta-analysis conducted by Woo et al. (2020) [7], which identified Southeast Asia and the Pacific regions as exhibiting the highest prevalence (13.7%) of nurse burnout. Moreover, a Malaysian national survey conducted in 2019 revealed an overall nurse burnout prevalence that was higher than the global average (24.4%) [11]. This finding was reinforced by Abd Wahab et al. (2023) [12], whose study highlighted a prevalence of work-related stress among healthcare professionals (HCPs), particularly nurses, at 24.3%.

Given the severity, many interventions have been taken at various levels to prevent and manage the nurse burnout epidemic. Addressing burnout in this population necessitates a comprehensive approach that encompasses both person- and organization-directed interventions [13]. Organization-directed interventions target specific systemic factors, such as modifying schedules, reducing workload intensity, enhancing teamwork and organizational culture, and increasing job control and resources [13]. While research has demonstrated the effectiveness of these interventions in reducing burnout [14,15], they face notable obstacles, including resistance to change [16], constraints on resources and leadership support [17], and challenges related to organizational culture [3]. In contrast, person-directed burnout interventions aim to enhance individual skills, resilience, and coping mechanisms, thereby providing immediate relief and improving job satisfaction and retention [16].

One such person-directed intervention, psychoeducation, involves educating individuals about mental health conditions, treatment options, and coping strategies through various formats such as face-to-face individual or group sessions as well as via online resources [18,19]. Psychoeducation interventions range from mindfulness, self-assessment, cognitive-behavioral techniques, rational emotive training, gratitude practice, meditation, and relaxation methods in the literature. These interventions empower nurses to manage their well-being and resilience by emphasizing intrinsic motivation and satisfaction. According to Pines (2000) [20], burnout reduction can be attained through the enhancement of coping skills such as cognitive stress management, relaxation techniques, effective time management, and social skills training. Nevertheless, the types of intervention may vary and different effect sizes have been reported [21].

While several reviews have been published on interventions to reduce nurse burnout, their scopes differ from ours. Notably, previous reviews have examined combined-strategy burnout interventions: Zang et al. (2020) [22] included both nurses and physicians, whereas Lee and Cha (2023) [23] focused specifically on nurses. Additionally, Hsu et al. (2024) [24] recently conducted a comprehensive evaluation of the efficacy of individual-based methods to reduce nursing burnout. Our review will examine a wider array of psychoeducational burnout interventions, including but not limited to mindfulness training. To the best of our knowledge, most systematic reviews of person-directed interventions focused exclusively on mindfulness training [25,26]. By providing a more comprehensive understanding of the available psychoeducational strategies, we hope to provide valuable insights for healthcare managers and policymakers to establish evidence-based burnout interventions that are effective in preventing and managing burnout among nurses.

## Objectives

Given the alarming prevalence of nurse burnout, it is crucial to identify effective psychoeducation-based interventions that can be implemented to mitigate burnout. This review aims to systematically examine and analyze prior research on person-directed psychoeducational interventions for burnout, assessing their impact and efficacy in mitigating burnout symptoms among nurses. Specifically, the review aims to address the following research questions:

1. What available person-directed psychoeducational burnout interventions are used across various healthcare settings to reduce nurse burnout levels?
2. Which of these interventions have been shown to effectively manage nurse burnout?

The findings will generate a comprehensive list of evidence-based burnout interventions that can be incorporated into modules specifically tailored to reducing nurse burnout. Moreover, the review will highlight sustainable and effective strategies for mitigating burnout. By thoroughly studying numerous psychoeducational interventions, assessing their efficacy in various nursing contexts, and identifying significant moderators and mediators that influence intervention outcomes, our systematic review aims to close this knowledge gap on how multiple strategies can synergistically reduce burnout symptoms among nursing personnel.

## Materials and methods

### Overview

This systematic review was conducted using the PRISMA (Preferred Reporting Items for Systematic Reviews and Meta-Analyses) checklist to ensure the quality and consistency of the procedure and reporting [S1 and S2 Tables]. A publicly available protocol was registered in the International Prospective Register of Systematic Reviews (PROSPERO) database. Additionally, the published protocol [27] acted as the blueprint for guiding the systematic exploration and mapping of the current literature concerning person-directed psychoeducational burnout interventions among nurses [S1 Data]. As this study involved a review of existing literature and did not involve human participants, ethical approval was not required.

### Search strategy

Five electronic research databases (PubMed-Medline, EBSCOhost, Ovid Medline, Scopus, and ScienceDirect) were searched. Our search strategy combined three key blocks of terms (burnout, nurses, burnout intervention) using Medical Subject Headings (MESH) terms, subject-specific headings, and keywords. Boolean operators (“AND” and “OR”) were applied to refine the search process. Examples of search strategies used for the databases are outlined in Table 1. In addition, a manual search for relevant articles was also conducted by examining the reference lists of articles included. Studies published in languages other than English and non-peer-reviewed journals were excluded. The search was limited to the ten years from January 1st, 2014, and December 31st, 2023.

**Table 1:** Examples of search strategies used for various databases.

Database	Search strategy
PubMed-MEDLINE	((((((((nurse*) AND (psychoeducation)) OR (coping)) OR (burnout intervention*)) OR (cognitive behavioural therapy)) OR (cognitive behavioural therapy)) OR (mindfulness)) OR (stress reduction)) AND (burnout)
Ovid MEDLINE	(((nurse*) and (psychoeducation or coping or burnout intervention* or cognitive behavioural therapy or cognitive behavioural therapy or mindfulness or stress reduction)) and (burnout)))
ScienceDirect	(“nurse”) AND (“psychoeducation” OR “coping” OR “burnout intervention” OR “cognitive behavioural therapy” OR “cognitive behavioural therapy” OR “mindfulness” OR “stress reduction”) AND (“burnout”)

In the initial stage, study titles and abstracts were screened, followed by a thorough examination of the full texts of the selected studies to determine the eligibility criteria. Searches, eligibility assessments, and data extraction were performed independently in an unblinded standardized manner by all team members working in pairs. Next, full-text appraisal was performed on selected articles before the list of included studies was finalized. Any discrepancies between reviewers at any stage of the record selection, data extraction, or appraisal process were first discussed between the paired reviewers to reach a consensus. If an agreement could not be reached, the issue was escalated to a broader team discussion involving all researchers. Final decisions were made through mutual agreement, ensuring methodological rigor and minimizing bias. This structured approach facilitated the inclusion of studies that best met the research criteria while maintaining the integrity and reliability of the review.

### Study selection

To facilitate the search strategy of this systematic review, the PICOS (Population, Interventions, Comparison, Outcome, Study Design) framework was employed to align the research question with corresponding search terms (Table 2). This review focused on the quantitative evaluation of person-directed psychoeducational interventions aimed at reducing burnout among nurses. Therefore, as a minimum requirement for inclusion, eligible studies must report the intervention outcome with validated evaluation tools using at least two time points. However, no restrictions were set for the practice setting of the nurses, be it hospital, community, or private practice. In terms of outcome, studies that did not directly report burnout among nurses, such as those focusing on depression, anxiety, or substance use were excluded. In addition, studies that evaluated organizational-directed burnout interventions such as changes to organizational policies or work procedures were not included. Only quantitative studies that involved case and control groups (randomized controlled trials, non-randomized experimental studies, and cohort studies) were included.

**Table 2:** Eligibility criteria based on the PICOS framework.

PICOS framework	Description
**P**opulation	Nurses working in healthcare settings across any country
**I**ntervention	All studies on interventions aimed at reducing levels of burnout among nurses in healthcare settings Interventions focused on person-directed psychoeducational approaches to address burnout
**C**omparison	An inactive control group that did not receive an intervention or received usual care, OR An active control group that received an alternative intervention for burnout
**O**utcomes	Characteristics of burnout interventions Changes in burnout levels from pre-intervention to post-intervention, including evaluation tools
**S**tudy design	Publications that are written in the English language with full text available from peer-reviewed journal articles Studies conducted between January 1st, 2014, and December 31st, 2023 Quantitative studies that involved case and control groups (randomised controlled trials, non-randomised experimental studies, and cohort studies)

### Data extraction

A data extraction sheet was developed using Google Sheets to extract data from the included studies. Criteria for data extraction were based on the inclusion and exclusion criteria. The primary outcome measure was burnout as measured by validated tools, either through researchers’ assessment or participants’ self-reporting. Summary data for each study included authorship, study design, participants, healthcare setting, intervention name, comparison group, measurement tools and overall outcomes, intervention details (name, type, activities, duration, mode of delivery, implementers, comparison group, follow-up), as well as burnout intervention outcomes were retrieved and synthesized descriptively.

### Quality assessment

All included articles were processed for the quality of analysis relevant to the research methodology. The Joanna Briggs Institute (JBI) critical appraisal tools were used to evaluate quantitative and quality evidence. The JBI critical appraisal tools are widely acknowledged as a reliable tool to investigate the study quality of various study designs such as RCT, systematic review, and observational studies.

### Data synthesis

During the data synthesis phase, all included studies were comprehensively examined, with study characteristics, quality, and intervention effects on nurse burnout tabulated in Google Sheets. A narrative synthesis approach was used to thematically organize and summarize all extracted information, identifying effective psychoeducational interventions for managing nurse burnout. Subgroup analyses were done to assess the differential effectiveness of interventions, categorizing them based on intervention types, such as online programs, in-person workshops, or blended learning formats to determine how delivery mode impacts effectiveness.

## Results

### Study inclusion

**Fig 1:**
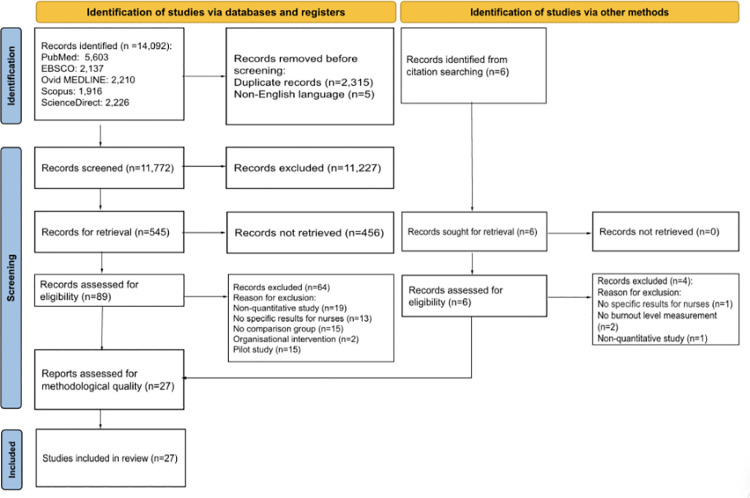
PRISMA flow diagram.

Fig 1 displays the results of the systematic review article selection process based on PRISMA guidelines. As the initial search strategy was purposefully broad and sensitive, the overall database search generated 14,092 records, with another six records identified from a secondary reference search. After title and abstract screening, 89 full-text articles were subjected to the inclusion and exclusion criteria. Following that, 64 articles were excluded for various reasons and only 27 studies met the criteria for final inclusion.

### Quality of the included studies

Table 3 outlines the detailed quality assessment results of all included articles using the JBI critical appraisal tool. We classified questions Q5 (“Were those delivering the treatment blind to treatment assignment?”), Q7 (“Were outcome assessors blind to treatment assignment?”) and Q9 (“Were outcomes measured in a reliable way?”) for randomized studies and question Q8 (“Were outcomes measured in a reliable way?”) for non-randomized studies as not applicable (NA) due to inherent methodological considerations specific to this type of research. The nature of psychoeducational interventions, which include interactive educational activities, makes it challenging to blind outcome assessors to treatment assignments. These activities are difficult to conceal from participants and facilitators, and primary outcomes are often measured using self-reported instruments, making participant blinding impractical. Additionally, the reliability of outcome measurements is upheld using standardized, validated tools like the Maslach Burnout Inventory (MBI) across the included studies, which have undergone rigorous psychometric validation, ensuring their reliability in accurately measuring burnout and its dimensions. Thus, the ‘NA’ classification for these questions acknowledges the specialized context of psychoeducational research and the established reliability of the measurement instruments used rather than indicating any oversight or disregard for the importance of these methodological aspects. Given their standardization and the robust methodological framework, they provide for evaluating subjective and self-reported outcomes specific to burnout, these tools inherently satisfy the reliability criterion. This approach also underscores our systematic review’s methodological rigor by aligning the measurement instruments’ proven reliability with the specific requirements for assessing psychoeducational interventions’ effectiveness in addressing nurse burnout.

**Table 3:** Quality appraisal using Joanna Briggs Institute critical appraisal tools.

JBI critical appraisal checklist for assessment of risk of bias for randomised controlled trials (n=13)														
Author(s), Year	Q1	Q2	Q3	Q4	Q5	Q6	Q7	Q8	Q9	Q10	Q11	Q12	Q13	Total
Özbaş & Tel (2015) [28]	Y^a^	N^b^	Y^a^	N^b^	NA^d^	N^b^	NA^d^	Y^a^	NA^d^	Y^a^	Y^a^	Y^a^	Y^a^	7/13
Wei et al. (2017) [29]	Y^a^	Y^a^	U^c^	U^c^	NA^d^	Y^a^	NA^d^	Y^a^	NA^d^	Y^a^	Y^a^	Y^a^	Y^a^	8/13
Grabbe et al. (2019) [30]	U^c^	U^c^	Y^a^	N^b^	NA^d^	Y^a^	NA^d^	Y^a^	NA^d^	Y^a^	Y^a^	Y^a^	Y^a^	7/13
Xie et al. (2020) [31]	Y^a^	Y^a^	Y^a^	U^c^	NA^d^	Y^a^	NA^d^	Y^a^	NA^d^	U^c^	Y^a^	Y^a^	U^c^	7/13
Huang et al. (2020) [32]	Y^a^	Y^a^	Y^a^	N^b^	NA^d^	Y^a^	NA^d^	Y^a^	NA^d^	Y^a^	Y^a^	Y^a^	Y^a^	9/13
Çelik & Kılınç (2022) [33]	Y^a^	U^c^	Y^a^	N^b^	NA^d^	Y^a^	NA^d^	Y^a^	NA^d^	Y^a^	Y^a^	Y^a^	Y^a^	8/13
Dahlgren et al. (2022) [34]	Y^a^	Y^a^	Y^a^	N^b^	NA^d^	Y^a^	NA^d^	Y^a^	NA^d^	Y^a^	Y^a^	Y^a^	Y^a^	9/13
Fong et al. (2022) [35]	Y^a^	Y^a^	Y^a^	Y^a^	NA^d^	Y^a^	NA^d^	Y^a^	NA^d^	U^c^	Y^a^	Y^a^	Y^a^	9/13
Pérez et al. (2022) [36]	U^c^	U^c^	Y^a^	N^b^	NA^d^	Y^a^	NA^d^	Y^a^	NA^d^	Y^a^	Y^a^	Y^a^	Y^a^	7/13
Laker et al. (2023) [37]	U^c^	Y^a^	U^c^	Y^a^	NA^d^	Y^a^	NA^d^	Y^a^	NA^d^	Y^a^	Y^a^	Y^a^	Y^a^	8/13
Lu et al. (2023) [38]	Y^a^	Y^a^	Y^a^	U^c^	NA^d^	Y^a^	NA^d^	Y^a^	NA^d^	N^b^	Y^a^	Y^a^	Y^a^	8/13
Sawyer et al. (2023) [39]	Y^a^	Y^a^	Y^a^	Y^a^	NA^d^	Y^a^	NA^d^	Y^a^	NA^d^	U^c^	U^c^	Y^a^	Y^a^	8/13
Sawyer, Tao & Bailey (2023) [40]	Y^a^	Y^a^	Y^a^	N^b^	NA^d^	Y^a^	NA^d^	Y^a^	NA^d^	N^b^	Y^a^	Y^a^	Y^a^	8/13
JBI critical appraisal checklist for quasi-experimental studies (non-randomised experimental studies) (n=14)														
Author(s), Year	Q1	Q2	Q3	Q4	Q5	Q6	Q7	Q8	Q9	Total				
Duarte & Pinto-Gouveia (2016) [41]	Y^a^	Y^a^	N^b^	Y^a^	Y^a^	N^b^	Y^a^	NA^d^	Y^a^	6/9				
Duarte & Pinto-Gouveia (2017) [42]	Y^a^	Y^a^	N^b^	Y^a^	Y^a^	Y^a^	Y^a^	NA^d^	Y^a^	7/9				
Slatyer et al. (2017) [43]	Y^a^	N^b^	N^b^	Y^a^	Y^a^	Y^a^	Y^a^	NA^d^	Y^a^	6/9				
Alenezi, McAndrew & Fallon (2019) [44]	Y^a^	Y^a^	N^b^	Y^a^	Y^a^	Y^a^	Y^a^	NA^d^	Y^a^	7/9				
Bagheri et al. (2019) [45]	Y^a^	U^c^	N^b^	Y^a^	Y^a^	Y^a^	Y^a^	NA^d^	Y^a^	6/9				
Kharatzadeh et al. (2019) [46]	Y^a^	Y^a^	N^b^	Y^a^	Y^a^	N^b^	Y^a^	NA^d^	Y^a^	6/9				
Luo et al. (2019) [47]	Y^a^	Y^a^	N^b^	Y^a^	Y^a^	Y^a^	Y^a^	NA^d^	Y^a^	7/9				
Franco & Christie (2021) [48]	Y^a^	Y^a^	N^b^	Y^a^	Y^a^	Y^a^	Y^a^	NA^d^	Y^a^	7/9				
Fu, Kao & Wang (2021) [49]	Y^a^	Y^a^	N^b^	Y^a^	Y^a^	Y^a^	Y^a^	NA^d^	Y^a^	7/9				
Hsieh et al. (2022) [50]	Y^a^	N^b^	N^b^	Y^a^	Y^a^	Y^a^	Y^a^	NA^d^	Y^a^	6/9				
Luo et al. (2023) [51]	U^c^	N^b^	N^b^	Y^a^	Y^a^	N^b^	Y^a^	NA^d^	Y^a^	4/9				
Othman, Hassan & Mohamed (2023) [52]	Y^a^	Y^a^	N^b^	Y^a^	Y^a^	Y^a^	Y^a^	NA^d^	Y^a^	7/9				
Safaeian et al. (2023) [53]	Y^a^	Y^a^	N^b^	Y^a^	Y^a^	N^b^	Y^a^	NA^d^	Y^a^	6/9				
Safavi, Kamrani & Asgharipour (2023) [54]	Y^a^	Y^a^	Y^a^	Y^a^	Y^a^	N^b^	Y^a^	NA^d^	Y^a^	7/9				

Y^a^ = yes.

N^b^ = no.

U^c^ = unclear.

NA^d^ = not applicable.

### Characteristics of included studies

Table 4 outlines the characteristics of the 27 studies in this review. Most were published in the second half of the search period. There were eight studies from 2023, five from 2019 and 2022 each, and two from 2020 and 2021, respectively. Geographically, there are six studies from China [28–33], followed by the United States of America (USA) [34–37] and Iran [38–41] with four studies each, as well as two from Portugal [42,43], Taiwan [44,45], and Turkey [46,47] respectively. The remaining countries, including Australia [48], Egypt [49], Hong Kong [50], Saudi Arabia [51], Spain [52], Sweden [53], and the United Kingdom (UK) [54], contributed one study each. Most of the studies (n=25) implemented burnout intervention programs in the hospital setting while the remaining two were held in specialized healthcare facilities, i.e., an elderly dementia institute and a mental health facility. Of the 27 studies, 13 applied a randomized controlled trial (RCT) design, while 14 were quasi-experimental. The sample size of study participants ranged from 46 to 296. In addition, the attrition rate of the participants ranged from 0% [28,49,52] to as high as 60.7% [34]. A total of 12 studies reported an attrition rate of <20%. However, four studies did not report the attrition rate [33,38,40,41].

**Table 4:** Characteristics of included studies (n=27).

Author(s), Year, Country	Design, Participants, Attrition Rate, Healthcare Setting	Intervention Name, Intervention Group (I^a^), Control Group (C^b^)	Measurement Tool	Main Outcome(s)
Özbaş and Tel [28], 2015, Turkey	RCT 82 (I=38, C=44) 18.7% Hospital	I^a^ - Psychodrama-based psychological empowerment programme C^b^ - No intervention	Psychological Empowerment Scale (PES)	Significantly higher psychological empowerment score at one- and three-months post intervention
			Nurse Work Empowerment Scale (NWES)	Significantly higher workplace empowerment score at three months post intervention
			Maslach Burnout Inventory (MBI)	Lower levels of emotional exhaustion (EE) and depersonalisation (DP) and higher level of personal accomplishment (PA) at one- and three-months post intervention
Duarte and Pinto-Gouveia [41], 2016, Portugal	Non-randomised controlled study 93 (I=45, C=48) 48.4% Hospital	I^a^ - Mindfulness-based group intervention C^b^ - Waitlist	Professional Quality of Life Scale-version 5 (ProQOL-5)	Significant decrease of compassion fatigue (CF) and burnout (BO) at post-test and three months
			Depression, Anxiety, Stress Scale (DASS-21)	Significant decrease in stress at post-test
			Acceptance and Action Questionnaire – II (AAQ-II)	Significant decrease in experiential avoidance at post-test
			Ruminative Responses Scale-Short (RRS)	No changes
			Five Facets of Mindfulness Questionnaire (FFMQ)	Significant increase in mindfulness post-test
			Self-Compassion Scale (SCS)	Significant increase in self-compassion post-test
Duarte and Pinto-Gouveia [(42)], 2017, Portugal	Non-randomised controlled study 93 (I=45, C=48) 48.4% Hospital	I^a^ - Mindfulness-based interventions (MBIs) C^b^ - Waitlist	ProQOL-5	Self-compassion significantly mediated the effects of the intervention on burnout, depression, anxiety, and stress symptoms
			DASS-21	
			SCS	
			AAQ-II	Psychological inflexibility significantly mediated the effects of the intervention of burnout, compassion fatigue, depression, and stress symptoms
			FFMQ	increased traits of mindfulness which mediated the effects of intervention on burnout, anxiety, stress, and satisfaction with life
			Satisfaction with Life Scale (SWL)	
Slatyer et al. [43], 2017, Australia	Non-randomised controlled study 91 (I=65, C=26) 36.3% Hospital	I^a^ - Mindful self-care and resiliency intervention (MSCR) C^b^ - Waitlist	ProQOL-5 Burnout subscale (BO)	Significant BO reduction between pre-intervention and six-month post intervention
			ProQol-5 Compassion Satisfaction subscale (CS)	Significant CS improvement post-test
			ProQol-5 Secondary Traumatic Stress subscale (STS)	Significantly lower STS at 6- month post intervention
			DASS-21	Statistically significant reductions in depression symptoms but no difference in stress and anxiety
			Connor-Davidson Resilience Scale-10 (CD-RISC10)	No significant improvement in resilience
			Generalised Self-Efficacy Scale (GSES)	No significant improvement in self-efficacy
			Self-Compassion Scale - Short Form (SCS-SF)	Significant increase in self-compassion post-test and six-month follow-up in intervention group
			WHO-5 Well-being Index (WHO-5)	Subjective quality of life scores significantly improved between pre and post and pre and follow-up in intervention group
Wei et al. [29], 2017, China	RCT 102 (I=51, C=51) 0% Hospital	I^a^ - Active intervention C^b^ - Regular management (focus group discussion (FGD) and luncheon parties)	Maslach Burnout Inventory - General Survey (MBI-GS)	EE and DP significantly decreased post-intervention
Alenezi, McAndrew and Fallon [44], 2019, Saudi Arabia	Quasi-experimental study 296 (I=154, C=142) 13.8% Hospital	I^a^ - Burnout prevention programme C^b^ - No intervention	MBI	Significant difference in burnout between intervention and control group in EE, DP, PA at 1-, 3-, 6- months Total burnout scores significantly lower in intervention group at 1- and 6-month EE, DP, and total burnout scores slightly increased at 3, and 6-month in both groups but intervention group had lower scores than control
Bagheri et al. [45], 2019, Tehran	Semi-experimental interventional study 60 (I=30, C=30) Not mentioned Hospital	I^a^ - Stress-coping strategies and group cognitive-behavioural therapy (CBT) C^b^ - No intervention	MBI	Burnout decreased immediately and significantly 1 month post intervention DP and PA decreased significantly immediately and 1 month later, but EE subscale did not show a significant decrease
Grabbe et al. [30], 2019, USA	Parallel RCT 196 (I=99, C=97) 60.7% Hospital	I^a^ -Community resiliency model (CRM) C^b^ - Nutrition/healthy eating class	WHO-5 Well-being Index (WHO-5)	Significant improvement in well-being post intervention up to one year
			CD-RISC	Significant improvement in resilience post intervention up to one year
			Secondary Traumatic Stress Scale (STSS)	Reduced in STS up to one year
			Copenhagen Burnout Inventory (CBI)	No significant burnout improvement over time
			Somatic Symptom Scale-8 (SSS-8)	Significantly reduced in somatic symptom up to one year
Kharatzadeh et al. [46], 2019, Iran	Experimental comparison trial 60 (I=30, C=30) 11.7% Hospital	I^a^ - Emotional regulation training (ERT) C^b^ - Waitlist	ProQOL-5	CS significantly increased and burnout significantly decreased post intervention No significant difference in CF scores
			Cognitive Emotion Regulation Questionnaire (CERQ)	Significant improvements in acceptance, refocusing on planning, positive refocusing, and positive reappraisal post intervention No significant differences in maladaptive strategies (self-blame, other-blame, rumination, and catastrophising)
			DASS-21	Significant reduction in depression, anxiety, and stress in the treatment group
Luo et al. [47], 2019, China	Quasi-experimental study 87 (I=41, C=46) 17.2% Hospital	I^a^ - Positive psychological interventions (PPIs) C^b^ - No Intervention	MBI-GS	Significant decrease in exhaustion post intervention No significant changes in cynicism or professional efficacy for both group
Huang et al. [32], 2020, China	RCT 152 (I=76, C=76) 3.9% Hospital	I^a^ - Balint group training C^b^ - No Intervention	MBI	Significantly reduced burnout in the intervention group particularly DP and EE PA scores increased slightly but not significantly post intervention
			Quality of Nursing Work Life Scale (QNWLS)	Significantly higher QNWLS scores post intervention Significant improvements in the work life-home life (WL-HL) and work world (WW) dimensions No significant changes in work context (WC) and work design (WD) dimensions
Xie et al. [31], 2020, China	RCT 106 (I=53, C=53) 15.2% Hospital	I^a^ - Mindfulness-based intervention on burnout group (MBIB) C^b^ - Education related to burnout group (EB)	MBI	Within groups: No significant changes in EE, DP, PA in EB group compared to significant improvements in MBIB group Between Groups: Significantly lower EE and DP scores, and higher PA scores in MBIB group than the EB group post-intervention
			Mindful Attention Awareness Scale (MAAS)	Within groups: No significant changes in MAAS scores in EB group but significant increase within MBIB group Between Groups: Significantly higher MAAS scores in the MBIB group post-intervention
			AAQ-II	Within groups: No significant changes in AAQ-II score of EB group over time but significant decrease in the MBIB group Between Groups: MBIB group showed significantly lower in AAQ-II scores post-intervention than the EB group
Franco and Christie [48], 2021, USA	Quasi-experimental study 53 (I=22, C=31) 9.4% Hospital	I^a^ - Self-compassion for healthcare communities (SCHC) C^b^ - No intervention	SCS	Significant increase SC from pre- to post-intervention and maintained at three-month follow-up
			Cognitive and Affective Mindfulness Scale (CAMS)	Significant increase in CAMS in the intervention group from pre- to post-intervention and maintained at three-month follow-up
			Compassion Scale (CFO)	Significant increase in CFO from pre- to post-intervention and maintained at three-month follow-up
			Professional Quality of Life (ProQOL)	Significant increase in CS from pre- to post-intervention and three-month follow up Significant decrease in BO scores from pre- to post-intervention and maintained at three-month follow-up No significant differences between group for STS
			DASS-21	Significant decrease in anxiety and stress scores from pre- to post-intervention and maintained at three-month No significant differences in depression scores
			Resiliency activation and decompression and job engagement	Significant increase in resiliency decompression at three-month follow-up No significant differences between groups for resiliency activation and job engagement.
Fu, Kao and Wang [49], 2021, Taiwan	Cluster experimental study 124 (I=67, C=57) 40.3% Hospital	I^a^ - 3R educational program intervention C^b^ - No Intervention	SF-12v2 Physical and Mental Health Summary Scale	Significant higher physical health (post-intervention) and mental health (post-intervention, one-month follow-up)
			ProQOL-5	Significant increase in CS (post-intervention, one- and three-months follow-up), lower BO (post-intervention, one-month follow-up), and lower STS (one- and three-months follow-up)
Çelik and Kılınç [33], 2022, Turkey	RCT 120 (I=60, C=60) 15.8% Hospital	I^a^ - Laughter yoga C^b^ - No Intervention	PSS	Significant decrease in perceived stress levels post-intervention in the intervention group
			MBI	Significant decreased in EE, DP and overall burnout with increased in PA post-intervention in the intervention group
			Life Satisfaction Scale	Significant increase in life satisfaction levels post-intervention in the intervention group
Dahlgren et al. [34], 2022, Sweden	Parallel RCT 207 (I=99, C=108) 37.2% Hospital	I^a^ - Proactive recovery programme C^b^ - Waitlist	Insomnia Severity Index (ISI)	No significant changes over time between the groups for insomnia symptoms, sleep quality, perceived stress, tension, listlessness, or beliefs and attitudes
			Karolinska Sleep Questionnaire (KSQ)	
			Dysfunctional Beliefs and Attitudes about Sleep Scale (DBAS-10)	
			PSS	
			Shirom-Melamed Burn-out Questionnaire (SMBQ)	Less burnout and fatigue symptoms post-intervention but not sustained at follow-ups
			Work Interference with Personal Life index (WIPL) from the Work Home Interference Scale	Work-induced fatigue decreased significantly post- test but not at follow-up
			Somatic Symptom Scale-8 (SSS8)	Somatic symptoms remained stable in the intervention group but increased in the control group, with significant differences between groups at post-test and follow-up
Fong et al. [35], 2022, Hong Kong	RCT 77 (I=39, C=38) 23.4% Hospital	I^a^ - Brief mindful colouring C^b^ - Waitlist	Perceived Stress Scale-10 items (PSS-10)	Intervention group effectively reduced perceived stress levels
			Short Warwick-Edinburgh Mental Well-being Scale (SWEMWBS)	No significant changes in mental well-being, burnout subscales, and trait mindfulness levels whether by per protocol analysis or intention-to-treat
				Maslach Burnout Inventory - Human Services Survey for Medical Professionals (MBI-HSS(MP))
				Five Facets of Mindfulness Questionnaire: Short Form (FFMQ-SF)
Hsieh et al. [50], 2022, Taiwan	Experimental study 80 (I=40, C=40) 1.3% Hospital	I^a^ - Gong meditation C^b^ - No Intervention	Occupational Burnout Inventory (OBI)	Significant difference in personal burnout scores between groups Significant reduction in personal, work-related, and client-related burnout at post-test Significant post-intervention reductions in all OBI subscales
			PSS	Significant difference in the PSS score between groups Significant reduction in perceived stress at post-test
Pérez et al. [36], 2022, Spain	RCT 74 (I=39, C=35) 0% Older people and dementia institution	I^a^ - Mindfulness training programme C^b^ - Waitlist	Spanish adaptation of ProQOL-5	Significant decrease in CF and burnout post-intervention and sustained at three-month No significant differences in satisfaction The effects of time and group comparison, after controlling for time, were statistically significant for all three subscales
Laker et al. [37], 2023, UK	Pragmatic RCT 198 (I=98, C=100) 56.1% Mental health organisation	I^a^ - Mind Management Skills for Life Programme C^b^ - Waitlist	Oldenburg Burnout Inventory (OLBI)	Significant improvement in burnout immediately after the intervention
			Warwick-Edinburgh Mental Well-being Scale (WEMWBS)	Significant improvement in well-being immediately after the intervention
Lu et al. [38], 2023, China	Parallel RCT 94 (I=47, C=47) 25.5% Hospital	I^a^ - Mindfulness-based program C^b^ - Health promotion strategies and inspirational quotes through *WeChat*	Maslach Burnout Inventory - Human Services Survey (MBI-HSS)	Significant reduction in EE post-intervention, sustained at two and six months Increased EE in control group over time
			Chinese-version of FFMQ	Significant improvement in mindfulness post-intervention, sustained at two-month
			Center for Epidemiological Studies Depression Scale (CESD)	Significantly decreased depression scores post-intervention, sustained for two months
			Zung Self-rating Anxiety Scale (SAS)	Significantly decreased anxiety scores post-intervention, sustained for two months
			Positive Affect and Negative Affect Schedule (PANAS)	Significantly improved subjective well-being scores post-intervention and maintained results for two months
				Satisfaction with Life Scale (SLS)
Luo et al. [51], 2023, China	Quasi-experimental study 130 (I=65, C=65) Not mentioned Hospital	I^a^ - Mindfulness decompression therapy programme C^b^ - Routine psychological nursing intervention	Symptom Checklist-90 (SCL-90)	Both groups showed decrease in all SCL-90 subscales post-intervention More significant improvement in the nucleic acid sampling area in the Intervention group with statistically significant reduction in all SCL-90 factors
			MBI-GS	Intervention group showed significant improvements in all burnout domains Professional efficacy of control group decreased post-intervention
Othman, Hassan and Mohamed [52], 2023, Egypt	Quasi-experimental prospective study 60 (I=30, C=30) 0% Hospital	I^a^ - MBIs C^b^ - No intervention	MBI-HSS (MP)	Significant improvements in all burnout domains
			FFMQ	Significant increase in overall mindfulness scores and improvements in four out of five mindfulness subscales (observing, describing, acting with awareness, and non-judging of inner experience)
			SCS	Significant increase in total self-compassion scores and improvements in five out of six self-compassion subscales (self-kindness, self-judgement, common humanity, isolation, and over-identification)
Safaeian et al. [53], 2023, Iran	Semi-experimental study 60 (I^1^=20, I^2^=20, C=20) Not mentioned Hospital	I^1c^ - Mindfulness training I^2d^ - Schema therapy C^b^ - Waitlist	Cognitive Fusion Questionnaire (CFQ)	Significant reduction in cognitive fusion scores for both interventions but schema therapy showed more pronounced effects than mindfulness training
			MBI	Both interventions significantly reduced burnout but mindfulness training showed a greater impact on reducing job burnout levels than schema therapy
Safavi, Kamrani and Asgharipour [54], 2023, Iran	Clinical trial study 46 (I=23, C=23) Not mentioned Hospital	I^a^ -Resilience training programs (RTPs) C^b^ - Virtual support session	CD-RISC	Significantly increased resilience scores post-intervention
			Maslach Burnout Questionnaire (MBQ)	Significantly decreased total burnout scores (significantly reduced EE, slightly reduced DP, and reduced PA) post-intervention Control group showed increased burnout scores (increased EE, DP and PA) post-intervention
Sawyer et al. [39], 2023, USA	Parallel RCT 75 (I=33, C=42) 34.7% Hospital	I^a^ - Resilience, insight, self-compassion and empowerment (RISE) C^b^ - Waitlist group	Brief Resilience Scale (BRS)	Significantly higher resilience scores at one-month and maintained at six-month
			Self-Reflection and Insight Scale (SRIS)	Significantly higher engagement in self-reflection at one-month No significant changes in need for self-reflection and insight subscales
			SCS-SF	Sustained improvements in self-compassion at one, three, and six months
			Psychological Empowerment Instrument (PEI)	No significant changes
			Stress Mindset Measure–General (SMM-G)	More positive stress mindset at one-month
			PSS	Significantly reduced perceived stress at one-month
			MBI	Significantly lower EE scores at one and three months Reduced DP scores at one-month No significant changes in PA
Sawyer, Tao and Bailey [40], 2023, USA	RCT 80 (I=40, C=40) 28.7% Hospital	I^a^ - RISE C^b^ - Waitlist group	Posttraumatic Growth Inventory (PTGI)	Significant improvements in post-traumatic growth at all follow-up points Significant improvements in subdomains of new possibilities, personal strength, and spiritual change
			ProQOL	Significant increase in CS scores post-test and one-month Significant reduction in BO scores post-test and one-month Significantly lower STS scores at one-month
			BRS	No significant changes
			SRIS	Significant increase in self-reflection and insight scores post-test and at one and three months
			SCS-SF	Self-compassion scores improved significantly at one, three, and six months
			PEI	Significant increase in overall psychological empowerment and its subdomains (meaning, competence, self-determination) post-test, one and three months
			GSES	No significant changes
			PSS	Significant decrease perceived stress at one month follow-up
			Brief Index of Affective Job Satisfaction (BIAJS)	No significant changes

I^a^ = intervention group.

C^b^ = comparison group.

I^1c^ = intervention group one.

I^2d^ = intervention group two.

In most studies, control groups involved a waitlist group (n=11) rather than an active control group (n=6). Furthermore, ten studies did not provide any intervention to the control group. Regarding the outcome measurement tools, the most frequently utilized instrument was Maslach’s Burnout Inventory (MBI) (n=15), followed by the Professional Quality of Life Scale version 5 (ProQOL-5) (n=8) and the Self-Compassion Scale (SCS) (n=6). The Five Facets of Mindfulness Questionnaire (FFMQ), Depression, Anxiety, Stress Scale (DASS-21), and Perceived Stress Scale (PSS) were also used in five studies each. In terms of outcomes, 15 articles concentrated on measuring burnout as the primary outcome [28–32,38–42,45,46,49–51]. About half (n=12) combined burnout with other outcomes such as perceived stress [35,36,47], compassion satisfaction [35,44], and mindfulness [35,50].

### Characteristics of burnout interventions

Table 5 describes the overview of burnout interventions in the 27 studies included in this review. The most applied intervention, whether as a single intervention or mixed with other strategies, was mindfulness-based interventions (MBIs) (n=16), followed by cognitive-behavioral therapy (CBT) (n=6). With regards to the mode of delivery, 14 out of 27 studies utilized physical group sessions solely. In contrast, seven studies relied only on a digital approach using materials such as compact discs (CDs), video, and online conferences. The remaining six studies applied a blended physical and digital delivery format. The duration of the interventions varied widely from one day to six months. Many of the studies practiced waitlist control groups (n=11) that received similar interventions later. Only six studies had an active control group that received similar treatment to the intervention group. The remaining 10 studies provided no intervention to the control groups. The outcome variable of interest, i.e., burnout, was measured at baseline (n=27), immediate post-intervention (n=24), and during one-month (n=8), two-month (n=2), three-month (n=11), six-month (n=7), and 12-month (n=1) follow-ups. A total of 10 studies measured burnout only at baseline and immediately post-intervention.

**Table 5:** Overview of burnout interventions.

Author(s), Year	Intervention Name, Type	Activities	Duration, Mode of delivery, Implementer, Comparison group	Follow-up Time Points
Özbaş and Tel, 2015 [28]	Psychodrama-based psychological empowerment programme Psychodrama-based Psychological Empowerment	Psychodrama sessions focusing on: Self -awareness Empathy Coping with stress Problem-solving Assertiveness training Exploration of past empowerment experiences and the concept of death Utilising role-play and staged scenarios to facilitate self-recognition and develop creative solutions Encouraging feedback and reflection on personal and professional experiences	10 weeks, 2 hours/session/week Face to face (group) Researcher (certified psychodramatist) No intervention	Pre, post, follow-up (1 month, 3 months)
Duarte and Pinto-Gouveia, 2016 [41]	Mindfulness-based group intervention Mindfulness-based interventions (MBIs)	Sitting meditation Mindfulness eating Body scan Breathing awareness and exercise Mindful communication exercise	6 weeks, 2 hours/session/week + 15 min/day Face to face (group) + compact discs (CD) (home practice) Clinical psychologist (MBSR trained) Waitlist group	Pre, post, follow-up (3 months)
Duarte and Pinto-Gouveia, 2017 [42]	Mindfulness-based intervention MBIs	Mindfulness of breath Bodily sensations Thoughts and sounds Meditation exercise	6 week, 2 hours/session/week + 15 min/day Face to face (group) + CD (home practice) Researcher (mindfulness-trained) Waitlist group	Pre, post
Slatyer et al., 2017 [43]	MSCR MBIs	Education workshop	1 full-day, 1.5 hours/4 sessions + 1.75 hours/week for 3 weeks (follow-up session) Face to face (group) + CD (home practice) Clinical psychologist (experience in running the intervention) Waitlist group	Pre, post, follow-up (6 months)
Wei et al., 2017 [29]	Active intervention Emotional regulation	Communication skills Conflict resolution approaches Efficacy elevation Emotion control Enhance working skills	6 months, 30 min twice/week Face to face (group) Nurse manager Regular management (FGD and luncheon parties)	Pre, post
Alenezi, McAndrew and Fallon, 2019 [44]	Burnout prevention programme CBT	Enhancing competencies, coping skills, and social support	2 days, 6 hours/day Face to face (group) Nurses (Master level - experienced and licensed in mental health nursing) No intervention	Pre, follow-up (1 month, 3 months, 6 months)
	Self-care Strategies	Deep breathing exercises Progressive muscle relaxation Stress reduction management		
	Communication and social skill training	Communication skills training Developing social support		
Bagheri et al., 2019 [45]	Stress-coping strategies and group CBT CBT	Identifying and recording thoughts Cognitive distortions recognition Behavioural consequences awareness Schema exploration and downward arrows Logical analysis and challenging beliefs Opposing negative beliefs Practical application and continuity planning	2.5 months, 1.5–2 hours/week for 10 sessions Face to face (group) Clinical psychiatrist (master’s degree) No intervention	Pre, post, follow-up (1 month)
Grabbe et al., 2019 [30]	CRM Sensory awareness techniques	Improved well-being, resiliency, secondary traumatic stress and physical symptoms Self-stabilisation during stressful work events	3 hours Face to face (group) 2 of the researchers (certified CRM Teachers) Nutrition/healthy eating class	Pre, follow-up (1 week, 3 months, 1 year)
Kharatzadeh et al., 2019 [46]	ERT Emotional regulation	Progressive muscle relaxation Non-judgemental awareness Acceptance and tolerance of emotional responses Modification of attention Cognitive reappraisal Problem solving Interpersonal skills	2 hours/6 sessions Face to face (group) Trained clinical psychologist Waitlist group	Pre, post
Luo et al., 2019 [47]	PPIs Gratitude exercise	Recording three good things Positive reflections Group support and encouragement	6 months, 5 times/week Social media - *WeChat* (group) Researcher No intervention	Pre, post
Huang et al., 2020 [32]	Balint group training Balint group training	Case presentations and discussions of challenging nurse-patient relationships	8 weeks, 1.5 hours/week Face to face (group) Senior Balint trainers No intervention	Pre, mid, post
Xie et al., 2020 [31]	MBIB MBIs	Body scan Mindfulness eating Mindful standing and lying yoga Sharing pleasant and unpleasant events Mindful walking Breathing exercise Loving-kindness and compassion meditation	8 weeks, 2.5 hours/week Face to face (group) Counsellor (engaged in psychological counselling for > 5 years and experience in leading mindfulness-based interventions for 3 years) Educational intervention	Pre, post, follow-up (1 month, 3 months)
Franco and Christie, 2021 [48]	SCHC Self-compassion	Writing compassionate notes to oneself Reflecting on core values	1 day, 1 hour/6 session (5 min break between session) Face to face (group) Not mentioned No intervention	Pre, post, follow-up (3 months)
	Mindfulness	Finding a supportive gesture Moments of mindfulness Self-compassion break Use of colouring supplies and small toys as diversion upon feeling overwhelmed		
	Resilience	Group discussion and reflection Booklet of concepts and practices from each session		
Fu, Kao and Wang, 2021 [49]	3R educational program intervention Resilience	Self-regulation through muscle relaxation Intentionality to modify impulsive thinking Perceptual maturation/self-validation to change one’s mood in stressful situations Connections and support through interaction with support networks Self-care and revitalisation through aerobic exercise, appropriate dietary and sleep regimens.	4 weeks, 2 hours/session/week Face to face (group) Researcher and psychologist No intervention	Pre, post, follow-up (4 weeks, 12 weeks)
	Mindfulness	Breathing exercises - practice and record exercise frequency and feelings in a notebook		
	Support	Organise own support network and write down the style, members, and functions of this network in their notebook		
Çelik and Kılınç, 2022 [33]	Laughter yoga Mindfulness	Deep-breathing exercises Warm-up exercises: clapping and body movements Childish games Laughter exercises followed by relaxation techniques	4 weeks, 1 hour/session/twice a week for 8 sessions Online through *Zoom* + *WhatsApp* (group) Not mentioned No intervention	Pre, post
Dahlgren et al., 2022 [34]	Proactive recovery programme CBT	Sleep formula Analysis of Behavior in Stressful Work Situations Unwinding Routines Before Bedtime Mindfulness and Body Scan Exercise Cognitive, Physical, and Emotional Fatigue Management Promoting Sleep According to Homoeostatic and Circadian Factors	4 weeks, 2.5 hours/session every second weeks for 3 sessions Face to face (group) Certified psychologist and Bachelor of applied psychology Waitlist group	Pre, post, follow-up (6 months)
	Motivational interviewing techniques	Encouragement to Reflect Group Discussions and Exercises Encouragement to Try New Strategies Reflection on Experience		
Fong et al., 2022 [35]	Brief mindful colouring MBIs	Colouring activities Mindfulness promotion Reflection and sharing experience	10 days, 20 min/5 working days Video (individual) Researcher Waitlist group	Pre, post
Hsieh et al., 2022 [50]	Gong meditation Sound therapy	Gong Meditation	2 days, 50–60 min/7 sessions (60 min break between session) Face to face (group) Qualified gong therapist No intervention	Pre, post
Pérez et al., 2022 [36]	Mindfulness training programme MBIs	Based on Kabat Zinn’s protocol Videos and interactive exercises of: Brief relaxation and breathing technique Content of the session Quote for personal reflection on topic covered Individual reflective writing exercise Audio-guided meditation audio Email address and phone number for general and technical support	6-week, 60 min/session/week Online through *Moodle* - video + audio (individual) Nurse and a psychologist (mindfulness-trained) Waitlist group	Pre, post, follow-up (3 months)
Laker et al., 2023 [37]	Mind Management Skills for Life Programme Mind management skills for life programme	Understanding the mind based on neuroscience and psychology Emotional skills management Practical strategies and skills development: Self-understanding and management of behaviours, thinking, and emotions Interactions with others Developing effective communication skills Managing one’s environment to support mental health and well-being Strategies for stress-free lifestyle. Personal functioning to build robustness and resilience Supplemental written materials and practical exercises: Establishing reflective practice Recognizing and managing unhelpful thoughts and behaviours Gaining insights into unconscious processes Developing effective communication Facilitated Group Discussions	8 weeks, 90 min/session/week Face to face (group) Trained and experienced facilitators Waitlist group	Pre, post^1^, post^2^, follow-up (6 months)
Lu et al., 2023 [38]	Mindfulness-based program MBIs	Loving-kindness meditation Mindfulness meditation Yoga exercise Group-sharing experiences	1 month, 2 hours/8 sessions + 2 hours retreat + 20 min/day Face to face (group) + *WeChat* and audio (home practice) Researcher (mindfulness-trained) Health promotion strategies and inspirational quotes through *WeChat*	Pre, post, follow-up (2 months, 6 months)
Luo et al., 2023 [51]	Mindfulness decompression therapy programme Mindfulness decompression therapy	Mindful eating Body scanning Mindful breathing Mindfulness meditation Mindfulness yoga Mindful walking Sitting meditation Self-exploration	8 weeks, 2 hours/week + 15–30 min/day at home Face to face (group) + *WeChat group* and audio (home practice) Certified psychological counsellors Routine psychological nursing intervention	Pre, post, follow-up (8 weeks)
Othman, Hassan and Mohamed, 2023 [52]	MBIs MBIs	Mindfulness eating Sitting meditation Mindful breathing Body scan Loving-kindness meditation Conscious movement Walking exercise Mindful listening exercise	2 months, 2.5 hours/8 sessions Live-streamed on *Zoom* (group)+ *WhatsApp group* (home practice) Researcher (mindfulness-trained) No intervention	Pre, post
Safaeian et al., 2023 [53]	Mindfulness training and schema therapy Mindfulness training	Based on Kabat-Zinn protocol Focused on: Stress factors Impact of thoughts and emotions on stress Practising formal meditation Performing daily activities mindfully	8 sessions, 60 min/session Face to face (group) + CD (home practice) Not mentioned Waitlist group	Pre, post, follow-up (2 months)
	Schema therapy	Based on protocol by Young et al. Integrated therapy to address early maladaptive schemas and underlying cognitive schemas that may contribute to job inefficiency		
Safavi, Kamrani and Asgharipour, 2023 [54]	RTPs Acceptance and commitment therapy (ACT) CBT Mindfulness-based cognitive therapy MBSR	Resilience and its components Communication and empathy skills Identifying personal capabilities Coping skills Anger management skills Teaching anxiety coping skills Promoting self-care practices Review and overview of previous sessions	6 sessions, 1 hour/session Virtual training class on CD (individual + home practice) Trained psychiatric assistant Virtual support session	Pre, post
Sawyer et al., 2023 [39]	RISE ACT CBT Mindfulness	Introduction session: guidelines and framework drivers and symptoms of burnout mindfulness Resilience session: stress recovery and oscillation coping skills connecting to joy and purpose Insight session: cognitive awareness emotional literacy Self-compassion sessions: self-compassion skills compassion in nursing compassion fatigue and secondary trauma self-validation combating negative self-talk and self-criticism Empowerment sessions: personal empowerment environmental impact on empowerment learned helplessness healthy boundaries authentic living values-behaviour alignment assertive communication self-advocacy Closing session: synthesis of learning self-care guide	8 weeks, 90 min/session/week Face to face (group) Licensed mental health counsellor and educator Waitlist group	Pre, follow-up (1 month, 3 months, 6 months)
Sawyer, Tao and Bailey, 2023 [40]	RISE ACT CBT Mindfulness	Introduction session: group guidelines program framework drivers and symptoms of burnout Resilience sessions: personal coping resources oscillation between stress and recovery post-traumatic growth connecting to purpose and meaning Insight sessions: cognitive and emotional awareness Self-compassion session: compassion fatigue and satisfaction self-compassion skills Empowerment sessions: healthy boundaries authentic living values-behaviour alignment Closing session: synthesis of learning self-care planning guide Authentic leadership concepts: relational transparency self-awareness values-behaviour alignment psychological flexibility	9 weeks, 90 min/week Virtual synchronous group sessions using Microsoft Teams Licensed mental health counsellor Waitlist group	Pre, post, follow-up (1 month, 3 months, 6 months)

The most prevalent type of intervention among the articles was single interventions (n=19) that involved various activities. The most implemented single intervention was MBIs, which included activities such as mindful breathing exercises, meditation, and self-care practices. Regarding mixed interventions, the most combined approaches were MBIs with CBT (n=3). Activities such as cognitive and emotional awareness, breath and sensory awareness, mindful exercise (movement, listening, meditation), empowerment, and resilience building were included in the mixed interventions. Commonly, those carrying out the interventions were mindfulness-trained researchers, certified clinical psychologists, and licensed mental health counselors.

### Outcomes of burnout interventions

Next, Table 6 provides a summary of the effects of burnout intervention. Out of 27 studies, 24 reported successful reductions in nurse burnout. Notably, nine studies demonstrated a highly significant effect (p<0.001) in reducing burnout at various stages. Out of these nine studies, six utilized single-focused interventions such as MBIs [31,33,47,49], psychodrama-based psychological empowerment [46], and sound therapy [45]. The remaining three studies [41,44,51] incorporated a comprehensive intervention approach consisting of mixed interventions such as cognitive-behavioral therapy (CBT), self-care strategies, and communication and social skill training. Successful burnout interventions consistently resulted in significant reductions in EE and DP scores, alongside an increase in PA scores. While most studies focused on the three main domains of burnout (EE, DP, PA), some also explored other outcomes related to secondary traumatic stress (STS) and compassion satisfaction (CS) [35,44,48]. On the other hand, three studies did not document significant decreases in any burnout dimensions following the intervention, either among the intervention groups individually or when compared to control groups [34,50,53].

**Table 6:** Summary of intervention and outcome on burnout.

Author(s), Year	Type of Intervention	Measurement tool for burnout	Outcome on burnout
Özbaş & Tel, 2015 [28]	• Psychodrama-based psychological empowerment	MBI (EE, DP, PA)	**Between the intervention and control groups:** **1-month follow-up:** • EE = ↓^₀^ • DP = ↓^c^ • PA = ↑^c^ **3-month follow-up:** • EE = ↓^c^ • DP = ↓^c^ • PA = ↑^c^ **Within intervention group:** • EE = ↓^c^ • DP = ↓^c^ • PA = ↑^c^
Duarte & Pinto-Gouveia, 2016 [41]	• MBIs	ProQOL-5 (BO, CS, STS)	**Time:** • BO = ↓^b^ • CS = ↑ • STS = ↓^b^ **Time x Group:** • BO = ↓ • CS = ↑ • STS = ↓^a^
Duarte & Pinto-Gouveia, 2017 [42]	• MBIs	ProQOL-5 (BO)	**Not reported p-value.** **Between intervention and control groups:** **Post:** • BO = ↓ (95% CI [−1.33 to −.12])
Slatyer et al., 2017 [43]	• MBIs	ProQOL-5 (BO, CS, STS)	**Group:** • BO, STS = ↓ • CS = ↑ **Time:** • BO = ↓ • CS = ↑ • STS = ↓^c^ **Time x Group:** • BO =↓^b^ • CS = ↑ • STS = ↓ **Within intervention group:** **Post:** • BO = ↓^c^ • CS = ↑^a^ • STS = ↓ **6-month follow-up:** • BO = ↓^b^ • CS = ↑ • STS = ↓^b^
Wei et al., 2017 [29]	• Emotional regulation	MBI-GS (EE, DP, PA)	**Between intervention and control groups:** **Post:** • EE = ↓^a^ • DP **=** ↓^a^ • PA = ↑
Alenezi, McAndrew & Fallon, 2019 [44]	• CBT • Self-care strategies • Communication and social skill training	MBI (EE, DP, PA)	**Between the intervention and control groups:** **1-month follow-up:** • EE = ↓^c^ • DP = ↓^c^ • PA = ↓^c^ **3-month follow-up:** • EE = ↓^c^ • DP = ↓^c^ • PA = ↓^c^ **6-month post follow-up:** • EE = ↓^c^ • DP = ↓^c^ • PA = ↓^c^
Bagheri et al., 2019 [45]	• CBT	MBI (EE, DP, PA)	**Between intervention and control groups:** **Post:** • Total burnout = ↓^b^ • EE = ↓ • DP = ↓^b^ • PA = ↓^a^ **1-month follow-up:** • Total burnout = ↓^b^ • EE = ↓ • DP = ↓^b^ • PA = ↓^a^
Grabbe et al., 2019 [30]	• Sensory awareness techniques	CBI (Work-related burnout (WRB))	**Time:** • WRB = ↓ **Intervention:** • WRB = ↓ **Time x Intervention:** • WRB = ↓
Kharatzadeh et al., 2019 [46]	• Emotional regulation	ProQOL-5 (BO, CS, STS)	**Between group:** **Post:** • BO =↓^a^ • CS = ↑^a^ • CF = ↓
Luo et al., 2019 [47]	• Gratitude exercise	MBI-GS (EE, Cynicism, Professional Efficacy (PE))	**Within intervention group:** **Post:** • EE = ↓^a^ • Cynicism = ↑ • PE = ↓
Huang et al., 2020 [32]	• Balint group training	MBI (EE, DP, PA)	**Time:** • EE = ↓^b^ • DP = ↓^b^ • PA = ↑^a^ **Group:** • EE = ↓^b^ • DP = ↓^b^ • PA = ↑ **Time x Group:** • EE = ↓^b^ • DP = ↓^b^ • PA = ↑
Xie et al., 2020 [31]	• MBIs	MBI (EE, DP, PA)	**Within intervention group:** **1-week post:** • EE = ↓^c^ • DP = ↓^c^ • PA = ↑^b^ **1-month follow-up:** • EE = ↓^b^ • DP = ↓^c^ • PA = ↑^c^ **3-month follow-up:** • EE = ↓^b^ • DP = ↓^c^ • PA = ↑^c^ **Between intervention and control groups:** **1-week post:** • EE = ↓^c^ • DP = ↓^c^ • PA = ↑ **1-month follow-up:** • EE = ↓^c^ • DP = ↓^c^ • PA = ↑^c^ **3-month follow-up:** • EE = ↓^c^ • DP = ↓^c^ • PA = ↑^c^
Franco & Christie, 2021 [48]	• Self-compassion • Mindfulness • Resilience	ProQOL (BO, CS, STS)	**Between the intervention and control groups:** **Time:** • BO = ↓^b^ • CS = ↑^a^ • STS = ↓ **Within intervention group:** **Time:** • BO = ↓^c^ • CS = ↑^a^ • STS = ↓
Fu, Kao & Wang, 2021 [49]	• Resilience • Mindfulness • Support	ProQOL-5 (BO, CS, STS)	**Between the intervention and control groups:** **Post:** • BO = ↓^c^ • CS = ↑^c^ • STS = ↓ **1-month follow-up:** • BO = ↓^a^ • CS = ↑^c^ • STS = ↓^a^ **3-months follow-up:** • BOt = ↓ • CS = ↑^c^ • STS = ↓^a^ **Within intervention group:** **Post:** • BO = ↓^c^ • CS = ↑ • STS =↓^c^ **1-month follow-up:** • BO = ↓^c^ • CS = ↑ • STS = ↓^c^ **3-months follow-up:** • BO = ↓^c^ • CS = ↓ • STS = ↓^c^
Çelik & Kılınç, 2022 [33]	• Mindfulness	MBI (EE, DP, PA)	Between the intervention and control groups: Post: • EE = ↓^c^ • DP = ↓^c^ • PA = ↑^c^ Within intervention group: Post: • EE = ↓^c^ • DP = ↓^c^ • PA = ↑^c^
Dahlgren et al., 2022 [34]	• CBT • Motivational interviewing techniques	SMBQ	Time: • Global score = ↓ • Fatigue = ↓ • Cognitive weariness = ↓ Group: • Global score = ↑ • Fatigue = ↑ • Cognitive weariness = ↑ Time x Group: • Global score = ↓^a^ • Fatigue = ↓^a^ • Cognitive weariness = ↓^a^
Fong et al., 2022 [35]	• MBIs	MBI-HSS (MP) (EE, DP,PA)	**Between intervention and control groups (intention to treat analysis):** **Post:** • EE = ↓ • DP = ↑ • PA = ↑ **Between intervention and control groups (per protocol analysis):** **Post:** • EE = ↓ • DP = ↑ • PA = ↑
Hsieh et al., 2022 [50]	• Sound therapy	OBI (Personal Burnout (PB), WRB, Client Related Burnout (CRB), Over-Commitment to Work (OCW))	**Within intervention group** **Post:** • PB = ↓^c^ • WRB = ↓^a^ • CRB = ↓^c^ • OCW = ↑ **Between intervention and control groups** **Post:** • PB = ↓^c^ • WRB = ↓^c^ • CRB = ↓^a^ • OCW = ↓^a^
Pérez et al., 2022 [36]	• MBIs	ProQOL-4 (BO, CS, CF)	**Between intervention and control groups** **Post:** • BO **=** ↓^a^ • CS = ↓ • CF = ↓^a^ **3-month follow-up:** • BO **=** ↓^a^ • CS = ↓ • CF = ↓^b^
Laker et al., 2023 [37]	• Mind management skills for life programme	OLBI	**Post intervention:** **(Time 2: After training group 1 intervention):** • OLBI Total = ↓^c^ • OLBI Disengagement = ↓^c^ • OLBI Exhaustion = ↓^c^ **(Time 3: After training group 2 waitlist control):** • OLBI Total = ↓ • OLBI Disengagement = ↓ • OLBI Exhaustion = ↓ **(Time 4: 6-month follow-up):** • OLBI Total = ↓ • OLBI Disengagement = ↓ • OLBI Exhaustion = ↓
Lu et al., 2023 [38]	• MBIs	MBI-HSS (EE, DP, PA)	**Intervention:** • EE = ↓^c^ • DP = ↓ • PA = ↑ **Time:** • EE = ↓^c^ • DP = ↓^a^ • PA = ↑ **Time x Intervention:** • EE = ↓^b^ • DP = ↓ • PA = ↑ **Between intervention and control groups:** **Post:** • EE = ↓^c^ **2-months follow-up:** • EE = ↑^c^ **6-months follow-up:** • EE = ↑^a^
Luo et al., 2023 [51]	• indfulness decompression therapy	MBI-GS (EE, Cynicism, PE)	**Between the intervention and control groups:** **Post:** • Total burnout = ↓^c^ • EE = ↓^c^ • PE = ↑^c^ • Cynicism = ↓^c^ **Within intervention group:** **Post:** • Total burnout = ↓^c^ • EE = ↓^c^ • PE = ↑^c^ • Cynicism = ↓^c^
Othman, Hassan & Mohamed, 2023 [52]	• MBIs	MBI-HSS (MP) (EE, DP, PA)	**Within intervention group:** **Post:** • EE = ↓^c^ • DP = ↓^b^ • PA = ↑^c^ **Between intervention and control groups:** **Post:** • EE = ↓^c^ • DP = ↓^c^ • PA = ↑^c^
Safaeian et al., 2023 [53]	• Mindfulness training • chema therapy	MBI	**Time:** • Total Burnout = ↓^b^ **Group:** • Total Burnout = ↓^b^ **Time x Group** • Total Burnout = ↓^b^
Safavi, Kamrani & Asgharipour, 2023 [54]	• ACT • CBT • Mindfulness-based cognitive therapy • MBSR	MBI (EE, DP, PA)	**Between the intervention and control groups:** **Post:** • Total Burnout = ↓^c^ • EE = ↓^b^ • DP = ↓ • PA = ↓^c^ **Within intervention group:** **Time:** • Total Burnout = ↓^c^ • EE = ↓^c^ • DP = ↓^b^ • PA = ↓^c^
Sawyer et al., 2023 [39]	• ACT • CBT • Mindfulness	MBI (EE, DP, PA)	**Between the intervention and control groups:** **1-month:** • EE = ↓^a^ • DP = ↓^a^ • PA = ↑ **3-month:** • EE = ↓^a^ • DP = ↓ • PA = ↓ **Within intervention group:** **1-months follow-up:** • EE = ↓ • DP = ↑ • PA = ↑ **3-months follow-up:** • EE = ↓ • DP = ↓ • PA = ↓ **6-months follow-up:** • EE = ↓ • DP = ↑ • PA = ↓
Sawyer, Tao & Bailey, 2023 [40]	• ACT • CBT • Mindfulness	ProQOL (BO, CS, STS)	**Within intervention group:** **Post:** • BO = ↓^a^ • CS = ↑^b^ • STS = ↓ **1-month follow-up:** • BO = ↓^b^ • CS = ↑^a^ • STS = ↓^a^ **3- and 6- months follow-up:** • BO, STS = ↓ • CS = ↑ **Between intervention and control groups:** **Post, 1-month and 3-month follow-up:** • BO, STS = ↓ • CS = ↑

^a^p<0.05.

^b^p<0.01.

^c^p<0.001.

### Effectiveness of burnout interventions over time

In addition, Table 7 compares the efficacy of single and mixed intervention strategies across various follow-up periods for the 24 studies that reported significant burnout reduction. Most single intervention strategies (n=16) followed nurses for less than three months (n=10), with only six studies following up to six months and none extending to 12 months or more. The majority were delivered physically (n=8), followed by combined physical and digital (n=4) and digital alone (n=4). Most single interventions, like MBIs, CBT, and emotional regulation, demonstrated short-term effectiveness, i.e., significant reductions in burnout symptoms immediately post-intervention and up to two months in some cases. As a single intervention, MBIs proved most effective when delivered through combined physical and digital modes, with sustained burnout reduction for up to six months [48]. However, a study by Lu et al. (2023) [32] that utilized a similar intervention showed that significant burnout reduction could only be sustained immediately post-intervention, with no difference in burnout symptoms observed when followed up to six months. The differences in effectiveness could be attributed to the activities used in the mindfulness interventions. On the other hand, the same intervention maintained effectiveness for three months when separately split into physical [31] or digital [52] formats. These findings suggest that single interventions can sustain effects for up to three months on physical or digital platforms [31,46,52], but a combined delivery mode is more effective for extending outcomes to six months [48].

**Table 7:** Studies with significant burnout reduction at different follow up time points (n=24).

Studies list according to intervention type and delivery mode	Burnout reduction at different follow-up time points^a,b^				
Post Intervention	1 month	2 months	3 months	6 months
**Single interventions**					
**Mindfulness-based interventions (n=8)**					
**Physical**					
Xie et al., 2020 [31]	√	√		√	
**Digital**					
Pérez et al., 2022 [36]	√			√	
Çelik & Kılınç, 2022 [33]	√				
Othman, Hassan & Mohamed, 2023 [52]	√				
**Combined**					
Duarte & Pinto-Gouveia, 2017 [42]	√				
Slatyer et al., 2017 [43]	√				√
Lu et al., 2023 [38]	√		x		x
Luo et al., 2023 [51]	√		√		
**Psychodrama-based Psychological Empowerment (n=1)**					
**Physical**					
Özbaş & Tel, 2015 [28]	√	√		√	
**Emotional regulation (n=2)**					
**Physical**					
Wei et al., 2017 [29]	√				
Kharatzadeh et al., 2019 [46]	√				
**CBT (n=1)**					
**Digital**					
Bagheri et al., 2019 [45]	√	√			
**Gratitude exercise (n=1)**					
**Digital**					
Luo et al., 2019 (EE only) [47]	√				
**Balint Group Training (n=1)**					
**Physical**					
Huang et al., 2020 [32]	√				
**Sound Therapy (n=1)**					
**Physical**					
Hsieh et al., 2022 [50]	√				
**Mind Management Skills for Life Programme (n=1)**					
**Physical**					
Laker et al., 2023 [37]	√				x
**MIXED INTERVENTIONS**					
**CBT + Self-Care Strategies + Communication and Social Skill Training (n=1)**					
**Physical**					
Alenezi, McAndrew & Fallon, 2019 [44]		√		√	√
**Self-compassion + Mindfulness + Resilience (n=1)**					
**Physical**					
Franco & Christie, 2021 [48]	√			√	
**Resilience + Mindfulness + Support (n=1)**					
**Physical**					
Fu, Kao & Wang, 2021 [49]	√	√		√	
**CBT + Motivational Interviewing Techniques (n=1)**					
**Physical**					
Dahlgren et al., 2022 [34]	√				x
**Mindfulness Training + Schema Therapy (n=1)**					
**Combined**					
Safaeian et al., 2023 [53]	√		√		
**Acceptance and commitment therapy (ACT) + CBT + Mindfulness-based cognitive therapy + Mindfulness-based stress reduction (n=1)**					
**Digital**					
Safavi, Kamrani & Asgharipour, 2023 [54]	√				
**ACT + CBT + Mindfulness (n=2)**					
**Physical**					
Sawyer et al., 2023 [39]		√		√	x
**Digital**					
Sawyer, Tao & Bailey, 2023 [40]	√	√		x	x

^a^√ = Significant burnout reduction.

^b^X = No significant burnout reduction.

In studies using mixed intervention strategies (n=8), six followed participants for 3–6 months post-intervention, while two had shorter follow-ups (<3 months). None extended follow-up to 12 months or more. Most interventions were delivered physically (n=5), with some using a combination of physical and digital approaches (n=1) or digital delivery solely (n=2). The most common interventions used in combination with others were MBIs and CBT. Of the four studies that applied CBT in person and tracked nurses for up to six months, only one study, which combined CBT with self-care, communication, and social skills training, demonstrated significant burnout reduction over the entire six-month follow-up period [51]. In contrast, three other studies that integrated CBT with MBIs as well as motivational, acceptance, and commitment therapies showed significant burnout reductions, but only for up to three months [36,37,53]. These findings suggest the importance of incorporating self-care and interpersonal skill enhancement to effectively reduce nurse burnout. Meanwhile, combining MBIs with interventions like self-compassion and resilience also led to significant burnout reduction for up to three months [35,44], while combination with approaches such as schema therapy, CBT, empathy skills training, and stress management showed effectiveness for up to two months [40,41]. However, the lack of long-term follow-up in these studies leaves uncertainty about sustained efficacy over longer periods.

A comparison between single and mixed intervention strategies showed that as a single intervention, MBIs yielded mixed outcomes when delivered via a combined physical and digital platform; one study showed results that lasted up to six months [48], while another study’s effects ended immediately post-intervention [32]. However, as a mixed intervention, MBIs combined with supportive practices can sustain positive results up to three months post-intervention [35,44]. Similarly, CBT when applied as a single intervention via a physical platform, was effective only for up to one month [38]. However, when CBT was combined with self-care and interpersonal skills training via a similar mode of delivery, the results were sustained for up to six months [51], indicating that combining certain therapeutic approaches can enhance and prolong the effectiveness of burnout interventions, making them more effective than single-intervention approaches.

## Discussion

### Principal findings

This review aimed to explore existing person-directed psychoeducational burnout interventions for alleviating burnout symptoms among nurses and analyze evidence on the effectiveness of these interventions. Of the 14,092 abstracts screened, 27 studies fulfilled the predetermined inclusion criteria. Based on the studies reviewed, psychoeducational interventions were found to be effective as both preventive and reactive tools for combating nurse burnout. This notion was also supported by a previous study that showed psychoeducational interventions as not only providing early measures to prevent burnout but also offering effective strategies for managing it once it manifests, underscoring the importance of a proactive approach to burnout management strategy [55]. Echoing prior research, psychoeducational interventions are instrumental not merely in relaying information but in fostering self-awareness and developing coping mechanisms that can safeguard long-term health and professional stability [56]. Additionally, they equip nurses with essential self-care skills and enhance resilience against workplace stressors, which also offer therapeutic benefits [16]. These findings highlight the comprehensive scope of psychoeducational interventions that encompass cognitive, emotional, and behavioral aspects of individuals.

Out of the 27 included studies in this review, 24 reported that person-directed psychoeducational interventions were significantly effective in reducing burnout symptoms. Additionally, six of these studies exhibited highly significant effectiveness at different stages of follow-up, with five implementing single types of intervention and only one study applying mixed types of intervention. The most common single intervention that significantly reduced nurses’ burnout was MBIs, which included activities like mindful breathing, meditation, mindful eating, and mindful exercises, all of which functioned to improve mental health, boost self-care, and enhance physical activity. As shown in previous studies, MBIs have been reported to significantly reduce stress, anxiety, and depression among nurses, notably when practicing Mindfulness-Based Stress Reduction (MBSR) [57]. In addition, mindful exercises used in MBIs also encourage nurses to be more physically active, resulting in reduced cortisol and increased endorphin levels, subsequently enhancing adaptive stress responses, improving mood and social skills, building resilience, reducing exhaustion, and regulating sleep [58–61].

Apart from that, this systematic review enhances the current literature on the effect of psychoeducational interventions on various domains of burnout. For instance, MBIs were found to significantly reduce emotional exhaustion and depersonalization and increase personal accomplishment. These findings were consistent with a previous meta-analysis that showed MBIs as effective interventions in reducing burnout [62]. Although burnout intervention effectively reduced emotional exhaustion and depersonalization, its impact on personal accomplishment was less straightforward. For instance, a meta-analysis published in 2016 found that improvement in reduced PA lasted only six months among nurses who received burnout intervention, as compared to improvement that lasted up to a year in EE and DP [63]. A similar observation was made by Brady et al. (2020) [64]. The lack of impact exerted by the burnout intervention on the domains of PA can be attributed to the problem-solving-centric components, such as empowering the participants with stress reduction and coping skills that are more beneficial for the domains of EE and DP [23]. However, a previous meta-analysis demonstrated that combining burnout interventions with others, such as organizational-directed interventions, significantly boosted personal accomplishment among HCPs, attributed to the synergistic effect that amplifies the overall positive impact of the intervention [65].

Furthermore, our systematic review demonstrates that combining multiple psychoeducational interventions significantly reduces nurse burnout through a holistic approach, providing a better solution than single interventions in tackling the complex nature of burnout. This result aligns with Aryankhesal et al. (2019) [66], which demonstrated notable burnout reductions in physicians and nurses using integrated methods. In addition, mixed interventions have also been shown to improve the physical and mental health of nurses [67]. In terms of the effectiveness duration, a previous study using mixed intervention to tackle burnout among inpatient elderly care nursing staff reported a longer burnout reduction of up to one year compared to only a short-term reduction at one month when using a single intervention [68].

Like previous studies, the most frequently used mixed intervention to alleviate burnout found in our systematic review was MBIs and CBT, with or without additional intervention [36,41,51]. As shown in the previous study, this synergy leverages their complementary strengths where mindfulness enhances present-moment awareness and non-judgmental acceptance of one’s experiences, which was vital for effectively engaging with CBT’s techniques of cognitive restructuring [69,70]. Meanwhile, behavioral modification in CBT helps counter maladaptive thinking and improve stress responses by identifying and challenging cognitive distortions and implementing strategies that promote adaptive coping mechanisms [71]. Furthermore, by combining mindfulness practices with cognitive restructuring, individuals can develop a more balanced perspective on stressors and enhance their coping skills. Research suggests that integrating mindfulness and CBT yields promising outcomes in addressing burnout, as first reported by a meta-analysis in 2010 [72] whereby interventions combining mindfulness and CBT produced large effects in reducing symptoms of burnout. Since then, several reviews [73,74] have also presented evidence that combined interventions are more effective in reducing rates of relapsed burnout.

The next important strategy commonly found used alongside other interventions to reduce burnout involves enhancing professional competency. This includes communication training that focuses on improving interpersonal skills and team and leadership communication. A previous study showed that positive communication skills were a good buffer of EE, besides promoting self-actualization [75] and enhancing resilience [76], ultimately reducing nurse burnout. Apart from individual benefits, enhanced communication skills also create a more supportive and efficient work environment, collectively reducing stress and burnout, and leading to better job satisfaction and overall well-being [77]. Other than effective communication, burnout intervention can also be a combination of other activities such as coping strategies, emotional regulation skills, and resilience, which were reported by Lee and Cha (2023) [23], to be effective in changing health professionals’ burnout to wellness. By combining different psychoeducational techniques, mixed interventions can tackle the multiple aspects of burnout more comprehensively.

Apart from the intervention components, the delivery mode of these psychoeducational interventions is also crucial in the effectiveness and sustainability of burnout management. Physical modes, such as face-to-face individual counselling and group therapy sessions, offer personalised support and are highly effective in providing emotional support and reducing feelings of isolation [78]. Our review found that the commonest delivery method for burnout interventions was via face-to-face group programs, similar to Lee and Cha [23]. Physical activities positively impact psychoneuroimmunology and mental health via changes in stress hormones [79]. However, the physical mode of delivery comes with its own set of challenges, such as the need to accommodate individual logistical and schedule preferences. Digital delivery methods, including web-based programs, virtual support groups, and online/offline self-help modules, offer flexibility and broader access. These web-based interventions have been reported to be effective in reducing symptoms of post-traumatic stress disorder (PTSD), depression, and anxiety, especially during the lockdown period of the COVID-10 pandemic [80]. The pandemic has accelerated the development of virtual encounters, but the downside of the digital mode of delivery was the difficulty in obtaining and sustaining participant engagement [81]. For instance, Barrett et al. (2021) [82] indicated that studies using web-based tools may have higher attrition rates in burnout intervention programs. Given the pros and cons of both modes, the combination of physical and online programs has been attempted by incorporating human interactions into digital interventions [83]. As per our findings, five studies demonstrated significant burnout reduction when digital modes of delivery were combined with physical sessions, such as Luo et al. (2023) [33] and Safaeian et al. (2023) [40], as supported by Ginoux et al. (2019) [84]. By deriving the benefits of both physical and digital modes of intervention, researchers and stakeholders can ensure optimal participant engagement and favourable outcomes [85].

The findings from this systematic review underscore the importance of comprehensive, multi-component psychoeducational interventions that can effectively address burnout among nurses and be sustained over a long time. Single interventions, such as MBSR and CBT often showed short-term efficacy but lacked long-term sustainability. For instance, in one study, MBSR delivered in physical and digital formats was effective for up to six months [48]. However, in another study, its effects were only limited to immediate post-intervention [32]. However, combining these single interventions with other supportive practices like self-compassion, resilience, and interpersonal skills training has demonstrated sustained burnout reduction for longer periods [35,44]. Combining CBT with self-care strategies and interpersonal skills training was also effective, with results sustained for up to six months [51]. Furthermore, pairing psychoeducation with psychotherapy has been shown to significantly boost the overall effectiveness of mental health care, deepening the understanding of personal well-being and adaptive strategies [86]. Given their multifaceted benefits, psychoeducational interventions are recommended as fundamental components of mental health strategies for mitigating burnout [56]. Nevertheless, despite this review demonstrating the potential of psychoeducational interventions to mitigate nurse burnout effectively, the wide diversity of approaches and their implementation across various modalities introduces variability that affects outcomes and poses challenges for synthesizing findings or establishing universal recommendations. This variability, including differences in intervention design, duration, delivery methods, and participant characteristics, can undermine the dependability of findings by making it difficult to compare results across studies or draw consistent conclusions on efficacy. This underscores the importance of tailoring interventions to specific contexts, such as addressing the unique stressors of specific environments or adapting programs to align with differing organizational cultures and resource availability.

Moreover, many studies included in this systematic review were limited to short-term follow-ups despite initial positive impacts, leaving the long-term sustainability of burnout interventions for nurses unclear. Aryankhesal et al. (2019) [66] highlighted the importance of prolonged follow-ups to determine the true long-term effects of these interventions. Repeated interventions might also help maintain reduced burnout levels over time, as suggested by Günüşen and Üstün (2010) [87]. Some studies failed to proceed with long-term follow-up due to high attrition rate [40] as well as constraints of human and financial resources [47,52]. Because of the cessation of follow-ups at specific time points, it could not be determined whether the observed lack of long-term effectiveness was real or simply a result of insufficient follow-up data. Therefore, likeminded researchers in this field should explore more systematic and feasible approaches to gauge long-term effects, as these interventions could possibly remain effective over time.

### Limitations

Several limitations emerged from this review and should be considered when interpreting the results. Many studies were conducted in single centers or specific regions, hence limiting the generalizability of the results to broader populations or different healthcare settings. Furthermore, most studies only assessed short-term effects up to three months post-intervention. The lack of long-term follow-up led to a suboptimal understanding of the sustained impact of burnout interventions. In addition, the studies analyzed in this review applied different instruments to measure burnout and could have resulted in heterogeneity. On top of that, the reliance on self-reporting to measure outcomes could have led to under or over-reporting of burnout symptoms depending on personal perceptions or social desirability, further affecting the accuracy of the results. Additionally, the studies generally lacked analysis of cultural factors, with interventions often designed without explicit consideration of sociocultural contexts, making it challenging to draw conclusions about the impact of cultural differences on the intervention effectiveness. Many studies used quasi-experimental designs (n=14), which are more prone to biases, such as selection bias and confounding factors, compared to randomized controlled trials (RCTs) [88,89], limiting the generalizability of findings and thus making it harder to determine the effects of the intervention. In addition, discrepancies in intervention format, training content, delivery methods, and follow-up duration further highlight the need to address contextual factors such as the target population’s needs, organizational environments, and cultural influences. While this review provides valuable insights into the efficacy of psychoeducational interventions, future research should aim to standardize intervention protocols, evaluation frameworks, and outcome measures. By doing so, interventions can be better designed to ensure consistent and effective results across diverse settings.

Moreover, implementation barriers may influence the outcomes of the test interventions. Only three studies described obstacles encountered in this review [36,51,54], including changes in hospital operations, challenges for participants in attending sessions due to time limitations, conflicting work schedules, increased workload, and the adjustment period required for transitioning to video-conference delivery instead of in-person sessions. These factors could have contributed to high attrition rates as well as diminished the reliability and validity of the findings. Based on the National Registry of Effective Prevention Programs’ study quality assessment scale, an attrition rate should be lower than 20% to be considered acceptable and favorable [90–92]. However, the attrition rates of the reviewed studies ranged from 0% [29,36] to 60.7% [30], with three studies not reporting attrition rates altogether [33,34,40]. Furthermore, most of the studies primarily focused on a few similar types of interventions, namely MBIs and CBT. There was also limited exploration of the delivery modes of the interventions, such as the impact of digital versus physical delivery. Such a narrow focus may have generated limited evidence about other potentially effective approaches.

The review process also had several limitations. It relied solely on electronic databases, potentially missing unpublished or grey literature, which might contain valuable but unpublished findings on burnout interventions among nurses. The review was restricted to English-language publications, which may have excluded relevant studies in other languages. Furthermore, by focusing on studies from the last decade, the review aimed to reflect current practices but may have introduced selection bias.

### Recommendations for future research

Based on our findings, several areas for future research can be considered to better understand the effectiveness of psychoeducational burnout interventions among nurses. To ensure long-term sustainability of the interventions, future research should emphasize longer follow-up periods, extending from six months to 12 months. A longer follow-up period allows a more comprehensive evaluation of the sustained impact of interventions; whether initial benefits are maintained, diminish, or evolve over time. All these are essential in providing a clearer understanding of the durability of intervention effects in real-world contexts. This also will assist in identifying any late-emerging effects or the need for booster sessions. Furthermore, evidence of sustained impact is critical to inform policy decisions and support the scaling-up of effective interventions, as short-term gains alone may be insufficient to justify long-term investment.

Apart from that, even though implementation of targeted interventions in high-pressure specialized settings such as Intensive Care Units and Oncology Units is particularly beneficial, future research should be expanded from single-center or localized regions to include diverse healthcare settings such as community health centers and specialized facilities in both rural and urban areas. A broader range of nursing populations such as those from different specialties, career stages, and cultural backgrounds can be recruited to enhance the generalizability and applicability of the interventions.

As mentioned in the limitation section, findings from existing studies included in this review may not fully capture cultural variability across burnout management. The importance of sociocultural contexts cannot be sidelined in future research. The development of culturally-sensitive interventions are more likely to align with the values, beliefs, and health practices of target populations, thereby improving their acceptability, relevance, and effectiveness. Recognizing and addressing sociocultural differences also helps identify potential barriers and facilitators to implementation and sustainability of these interventions. For this purpose, a qualitative research approach will be preferred as it can offer valuable insights into contextual nuances such as workplace norms, coping mechanisms, and attitudes toward mental health and burnout. Such research could complement quantitative findings and provide a more holistic understanding of how interventions can be tailored to fit diverse cultural contexts. A higher representation of low- and middle-income countries (LMICs) may enhance the understanding of how cultural and resource differences may influence the success of these interventions. Additionally, incorporating a combination of both subjective self-reports and objective measures, such as physiological indicators (e.g., cortisol levels) and performance metrics (e.g., absenteeism rates) may help to reduce potential bias. This multimodal outcome reporting approach can provide a more comprehensive assessment of intervention effectiveness.

Exploring a broader range of potentially effective psychoeducational interventions, such as narrative therapy, art therapy, resilience training, and peer support, alongside commonly studied methods like MBIs and CBT, can help identify new and innovative solutions for burnout reduction. Given the increasing use of technology in healthcare, flexible and accessible technology-enhanced interventions, such as mobile apps and telehealth, should be evaluated for their feasibility, acceptability, and effectiveness in comparison with traditional in-person methods. These intervention approaches may be more practical for nurses working in remote or resource-limited settings. This review also did not explicitly assess implementation fidelity, and the majority of included studies lacked sufficient reporting on key fidelity components, such as facilitator training, adherence monitoring, and quality assurance mechanisms. Consequently, a comprehensive synthesis and analysis of implementation fidelity across interventions was not feasible. Future studies should use standardized protocols and incorporate rigorous measures of implementation fidelity (e.g., observation checklists, adherence measures, and feedback mechanisms) to better assess the true impact of the interventions. More importantly, these components should be reported more consistently to enhance replicability of intervention delivery across diverse contexts.

Another area worth exploring is the development of personalized interventions customized to an individual’s unique needs based on machine learning algorithms to predict the most effective intervention components based on personal characteristics and work stressors. Furthermore, integrating these interventions with organizational measures has been shown to enhance their effectiveness and yield long-lasting benefits(85). This highlighted the benefit of simultaneously addressing multiple dimensions of burnout, making interventions more comprehensive and effective. Finally, implementation science frameworks should be applied in future research to identify barriers and facilitators to successful adoption and integration of the interventions into routine clinical practice. To secure buy-ins from stakeholders, economic evaluation is also the way forward to provide cost-related benefits for policymakers and healthcare administrators to make informed decisions about resource allocation for burnout management programs.

## Conclusion

The systematic review highlights the importance of addressing nurse burnout through psychoeducational interventions, revealing that both single and mixed approaches, especially MBIs and CBT, can effectively reduce burnout levels among nurses. However, the sustainability of the intervention remains a concern, necessitating long-term follow-up studies. Additionally, the review findings shed light on the benefits and challenges of different delivery modes, noting that while face-to-face interventions offer personalized support, digital and blended formats provide flexibility and broader access. Because of the varying intervention effectiveness based on the components and delivery modes of interventions, continued research and development of tailored, sustainable intervention modules are needed. Future research should focus on exploring a broader range of strategies across diverse healthcare environments with both objective and subjective measures such as cross-cultural differences to establish evidence-based and culturally appropriate interventions that can effectively improve nurse well-being, reduce turnover, and enhance patient care.

## Supporting information

S1 Table PRISMA 2020 abstract checklist. (PDF)

S2 Table PRISMA 2020 checklist. (PDF)

S1 Data Psychoeducational burnout intervention for nurses: protocol for a systematic review. (PDF)

S2 Data 14,098 Compiled list of records. (XLSX)

## Abbreviation3s:

AAQ-II: Acceptance and Action Questionnaire – II
ACT: Acceptance and Commitment Therapy
BO: Burnout
BRS: Brief Resilience Scale
CAMS: Cognitive and Affective Mindfulness Scale
CBI: Copenhagen Burnout Inventory
CBT: Cognitive-Behavioral Therapy
CD: Compact Discs
CD-RISC: Connor-Davidson Resilience Scale-10
CFO: Compassion Scale
CF: Compassion Fatigue
CRB: Client Related Burnout
CRM: Community Resiliency Model
CS: Compassion Satisfaction
DASS-21: Depression, Anxiety, Stress Scale
DP: Depersonalization
EB: Education related to Burnout group
EE: Emotional Exhaustion
ERT: Emotional Regulation Training
FFMQ: Five Facets of Mindfulness Questionnaire
GSES: Generalized Self-Efficacy Scale
HCP: Healthcare Professional
MBI: Maslach’s Burnout Inventory
MBI-GS: Maslach Burnout Inventory – General Survey
MBI-HSS: Maslach Burnout Inventory – Human Services Survey
MBI-HSS(MP): Maslach Burnout Inventory – Human Services Survey for Medical Professionals
MBIs: Mindfulness-Based Interventions
MBIB: Mindfulness-Based Intervention on Burnout group
MBSR: Mindfulness-Based Stress Reduction
MSCR: Mindful Self-Care and Resiliency intervention
OBI: Occupational Burnout Inventory
OLBI: Oldenburg Burnout Inventory
OCW: Over-Commitment to Work
PA: Personal Accomplishment
PB: Personal Burnout
PE: Professional Efficacy
PEI: Psychological Empowerment Instrument
PICOS: Population, Interventions, Comparison, Outcome, Study Design
PPIs: Positive Psychological Interventions
ProQOL: Professional Quality of Life
ProQOL-5: Professional Quality of Life Scale version 5
PSS: Perceived Stress Scale
QNWLS: Quality of Nursing Work Life Scale
RCT: Randomized Controlled Trial
RISE: Resilience, Insight, Self-compassion and Empowerment
RTPs: Resilience Training Programs
SCHC: Self-compassion for healthcare communities
SCL-90: Symptom Checklist-90
SCS: Self-Compassion Scale
SCS-SF: Self-Compassion Scale – Short Form
SMBQ: Shirom-Melamed Burn-out Questionnaire
SRIS: Self-Reflection and Insight Scale
STS: secondary traumatic stress
UK: United Kingdom
USA: United States of America
WHO-5: WHO-5 well-being index
WRB: work-related burnout
