# Supplementary material for: Person-directed burnout intervention for nurses: A systematic review of psychoeducational approaches
Source: PLoS One. 2025 May 9;20(5):e0322282. doi: 10.1371/journal.pone.0322282 (PMC12063909; doi:10.1371/journal.pone.0322282)
Supplement: S1 Data — (PDF) [file pone.0322282.s003.pdf]

Protocol

Psychoeducational Burnout Intervention for Nurses: Protocol for a Systematic Review

Ili Abdullah Sharin*, MD; Norehan Jinah*, MD; Pangie Bakit*, MBBS; Izzuan Khirman Adnan*, MBBS; Nor Haniza Zakaria*, MD; Shazwani Mohmad*, MD, MEL, MPH, PhD; Siti Zubaidah Ahmad Subki*, MD, MPH; Nursyahda Zakaria*, BSc, MSc; Kun Yun Lee*, MBBChBAO, MPH, DrPH

Centre of Leadership & Professional Development (CLPD), Institute for Health Management (IHM), National Institutes of Health (NIH), Ministry of Health Malaysia, Shah Alam, Malaysia

*all authors contributed equally

Corresponding Author:
Ili Abdullah Sharin, MD
Centre of Leadership & Professional Development (CLPD)
Institute for Health Management (IHM)
National Institutes of Health (NIH), Ministry of Health Malaysia
Block B1, NIH Complex, No. 1 Jalan Setia Murni U13/52
Section U13, Setia Alam
Shah Alam, 40170
Malaysia
Phone: 60 33628314
Email: ppilias@moh.gov.my

Abstract

Background: Nurses face high levels of stress and emotional exhaustion due to heavy workloads and demanding work environments. Prolonged exposure to these stressors predisposes nurses to burnout, which can adversely affect patient care. Addressing burnout among nurses requires a multifaceted approach, involving both personal and organizational strategies. While organizational strategies target systemic workplace issues, personal interventions are often favored for their ease of implementation, immediate benefits, and empowerment of health care workers through stress management and resilience-building. Prioritizing evidence-based interventions to mitigate burnout among nurses is crucial for managing occupational stress and promoting well-being. Person-directed psychoeducation is an effective personal intervention strategy used to equip nurses with the appropriate knowledge and skills to handle stressors, thereby safeguarding their mental health and ensuring high-quality patient care.

Objective: This protocol proposes a systematic review that aims to identify and assess the effectiveness of person-directed psychoeducational interventions for nurses. The review aims to pinpoint effective interventions that can be implemented to manage burnout and support the mental health of nurses.

Methods: This systematic review will follow the PRISMA (Preferred Reporting Items for Systematic Review and Meta-Analysis) guidelines. In total of 5 electronic databases (PubMed-MEDLINE, EBSCOhost, Ovid MEDLINE, Scopus, and ScienceDirect) will be searched for studies published between January 1, 2014, and December 31, 2023. The search will encompass 3 main keywords: “nurses,” “burnout intervention,” and “burnout.” Predefined eligibility criteria will guide the screening process. Data will be extracted to address the objectives of the review. The risk of bias for each study will be assessed using Joanna Briggs Institute Critical Appraisal Tools.

Results: Preliminary searches have been initiated since February 2024, with the review expected to be completed by June 2024. The expected results will include a comprehensive list of psychoeducational interventions and their effectiveness in reducing burnout among nurses. The review will highlight interventions that demonstrate significant impact in published studies from various countries.

Conclusions: Given the rising prevalence of burnout among nurses and its detrimental effects on individuals and health care organizations, the findings from this systematic review are expected to inform health care policy and practice. By evaluating different interventions, it will provide insights into the most effective strategies, contributing to evidence-based practices that support nurses’ mental health and well-being. The findings can support stakeholders in developing and implementing targeted

XSL•FO
RenderX

strategies to combat nurse burnout, ultimately enhancing the quality of patient care and health care delivery. In addition, the findings will also offer valuable information for researchers, guiding future practice and research in this area.

Trial Registration: PROSPERO CRD42024505762; https://tinyurl.com/4p84dk3d

International Registered Report Identifier (IRRID): DERR1-10.2196/58692

(JMIR Res Protoc 2024;13:e58692) doi: 10.2196/58692

KEYWORDS

burnout intervention; burnout; psychoeducation; nurse; systematic review; protocol; evidence-based intervention; effectiveness; psychoeducational intervention; mental health.

Introduction

Background

In recent decades, health care systems have undergone significant transformations characterized by increased complexity and heightened demands in patient care [1]. This evolution is marked by a surge in the severity of medical conditions experienced by patients in hospitals, necessitating a more intricate and intensive approach to their treatment [2]. Health Care Professionals (HCPs), the frontline professionals responsible for delivering medical care, find themselves immersed in a work environment that has become more challenging and fast-paced, necessitating them to enhance their skills and expertise to meet the escalating demands of medical practice. Consequently, the responsibilities undertaken by HCPs have multiplied, requiring them to juggle multiple facets of patient care with higher precision and efficiency [3]. Long working hours and heavy workloads have become the norm, placing an extraordinary burden on HCPs. As a result, the intense nature of their work has contributed to a pervasive issue known as burnout [4].

The *ICD-11* (*International Classification of Diseases, 11th Revision*), defined burnout as a syndrome stemming from chronic workplace stress that is not effectively managed. It encompasses feelings of exhaustion, depersonalization, and a decreased sense of personal accomplishment [5]. The dimension of exhaustion is described as a feeling of physical and emotional fatigue, leading to a significant decrease in energy levels due to prolonged work-related stress. Next, the depersonalization dimension involves a sense of detachment or disengagement from one’s work, often resulting in negative attitudes or cynicism toward work, colleagues, and the organization. Finally, the reduced sense of personal accomplishment refers to a perceived decline in competence and effectiveness in professional roles, often manifesting as diminished productivity and motivation. Burnout is different from stress and other psychological disorders because it involves the coexistence of these 3 elements. In addition, burnout is characterized by a prolonged experience of these symptoms, distinguishing it from stress [6].

Nurse burnout is a significant concern in health care settings. Work-related stress is a prominent factor contributing to this issue [7]. Nurses, often considered the backbone of health care provision, face high levels of stress and emotional exhaustion that can lead to burnout. The development of burnout in nurses is a complex issue influenced by various factors [8], including excessive workload, inadequate staffing, and interpersonal conflicts involving patients, guardians, and medical staff [9]. Furthermore, the lack of control over their work and decision-making with a need to provide efficient care in a limited timeframe can create a stressful environment that further predisposes them to burnout [10]. Moreover, the absence of good leadership support [11], and access to resources and guidance, can amplify stress levels and contribute to burnout among nurses.

Globally, numerous studies with varying burnout prevalence rates have been reported in different regions, highlighting the magnitude and severity of the problem worldwide. A systematic review conducted in 2013 found that burnout prevalence ranged from 22% to 40% among nurses in 10 European countries [12]. Asian countries were not spared of the same burnout epidemic among nurses. A meta-analysis conducted by Woo et al [13] revealed that the Southeast Asia and Pacific regions recorded the highest prevalence (13.7%) of burnout among all 6 global regions. A study conducted in Malaysia by Abd Wahab et al [14] highlights the prevalence of work-related stress among health care workers, particularly nurses, with a prevalence of 24.3%. Similarly, another national-level survey revealed that approximately 1 in 4 (24.4%) nurses in the public sector experienced burnout, with hospital nurses having a slightly higher prevalence (25.8%) than primary care nurses (19.3%) [15].

The consequences of nurse burnout are extensive [16], ranging from impact on individual physical and psychological well-being, job satisfaction, organizational commitment, and health care quality [17,18]. On a personal level, it can lead to increased stress, anxiety, depressive symptoms, reduced attention to detail, and impaired decision-making abilities, all of which can manifest as organizational issues such as increased medical errors, compromised care quality, and lower patient satisfaction [19,20]. In addition, absenteeism and high turnover rates from burnout can contribute to workforce shortages and economic implications for health care systems [21]. Hence, it is crucial to mitigate the burnout epidemic among HCPs, including nurses. Research has shown that effectively managing nurse burnout translates into significant economic benefits for health care systems. In a recent study, a hospital’s annual expenses associated with turnover attributable to nursing burnout were estimated to be US $16,736 per nurse. However, it dropped to US $11,592 per nurse annually at institutions with a burnout reduction program [22].

XSL•FO
RenderX

Tackling burnout among HCPs demands a multifaceted approach encompassing person- and organization-directed interventions. A holistic solution is often the most effective [23]. Organization-directed interventions target systemic factors such as workload and organizational culture within the work environment [24]. It is undeniable that organizational-directed interventions remain important in addressing systemic issues contributing to burnout and fostering a supportive work environment. Nevertheless, they are difficult to implement and maintain due to inherent challenges such as resistance to change and the complexity of altering organizational structures [25] as well as the need for strong evidence in terms of long-term benefits and sustainability [26].

Consequently, person-directed interventions emerge as a favorable option for addressing burnout among HCPs, especially nurses. Person-directed interventions enhance individual skills, resilience, and coping mechanisms by offering practical tools and strategies for HCPs to manage burnout proactively [24]. Some examples of these interventions include stress management techniques and mindfulness training [27] that empower individuals to recognize and address burnout symptoms early on. They are easier to implement and can yield immediate improvements in well-being by providing the right tools, techniques, and self-care strategies [28] and fostering a sense of personal agency in managing burnout. Prioritizing person-directed interventions in the absence of organizational-directed strategies can provide practical solutions to improving well-being and job satisfaction among HCPs [29]. A physically and emotionally healthier workforce that is better equipped to navigate the complexities of the health care landscape [30] can ultimately enhance patient outcomes and overall health care quality [31].

Review Questions

Burnout represents a significant challenge within the health care sector, particularly among nurses. Despite the critical need for effective burnout interventions, the optimal strategies for addressing nurses' burnout remain unclear, primarily due to inconclusive evidence on the effectiveness of specific burnout interventions for nurses. Although several reviews have been published on interventions to reduce nurse burnout, their target groups are different from ours. For instance, Zhang et al [32] focused on nurses as well as physicians, whereas Lee and Cha [33] targeted clinical nurses solely.

Our review will examine a wider range of psychoeducational burnout interventions, including but not limited to mindfulness training, in contrast to the reviews by Suleiman-Martos et al [34] and Sulosaari et al [35], both of which focused on the effects of mindfulness training on burnout among nurses. In addition, a recent comprehensive evaluation of the efficacy of individual-based methods to lessen nursing burnout was carried out by Hsu et al [36]. Our systematic review seeks to close this gap by offering a thorough study of numerous psychoeducational interventions, assessing their efficacy in various nursing contexts, and identifying significant moderators and mediators that affect intervention results. This approach will provide a more comprehensive understanding of how various strategies can be combined to synergistically reduce burnout symptoms among nursing personnel.

In response to this evidentiary gap, we aim to systematically review and analyze published studies on person-directed psychoeducational burnout interventions and evaluate their effectiveness in mitigating burnout symptoms among nursing staff. Guided by a comprehensive analytical framework, this investigation will address the following research questions: (1) What available person-directed psychoeducational burnout interventions are used across various health care settings to reduce nurses' burnout levels? (2) Which of these interventions has effectively managed burnout among nurses? (3) What are the challenges and facilitators of these interventions?

Ultimately, we aimed to provide a comprehensive overview of effective person-directed psychoeducational burnout interventions tailored to nurses based on existing research evidence. In addition, this review seeks not only to delineate effective strategies but also to identify gaps within the existing literature and areas that require further investigation in the context of psychoeducational burnout interventions for nurses in the current health care setting. Using an inclusive methodological approach, this review will critically evaluate a broad spectrum of studies and settings, ensuring a thorough assessment of intervention strategies.

Methods

Overview

The systematic review will adhere to the PRISMA (Preferred Reporting Items for Systematic Review and Meta-Analysis) 2020 checklist (Multimedia Appendix 1) [37] to ensure a rigorous and transparent approach. These guidelines provide a standardized framework for conducting systematic reviews, ensuring that the review process is comprehensive and replicable with minimal bias. The review protocol has been registered in the PROSPERO (International Prospective Register of Systematic Reviews) database (CRD42024505762) [38]. PROSPERO is an international database of prospectively registered systematic reviews, which provides a unique permanent registration number to the protocol that prevents duplication and promotes transparency, thereby reducing reporting bias. The final review will be reported following the PRISMA statement [39]. Necessary amendments to this protocol will be reported and published with the review results.

Search Strategy

To ensure a thorough and comprehensive literature retrieval, we will screen 5 electronic bibliographic databases, that consist of PubMed-MEDLINE, EBSCOHost, Ovid MEDLINE, Scopus, and ScienceDirect. These databases are known for their comprehensive inclusion of medical and scientific publications. Our search strategy, designed for maximal sensitivity, will leverage a meticulously curated combination of Medical Subject Headings terms, subject-specific headings, and keywords. These terms are strategically chosen to encapsulate core concepts and variables pertinent to our research inquiry. Furthermore, Boolean operators ("AND" and "OR") will be used to refine the information retrieval process. These operators allow for the

XSL•FO
RenderX

combination of different search terms, enabling precise modification of the search scope to either expand or narrow down the results according to the specific requirements of the study.

The PICO (Population, Intervention, Comparison, and Outcomes) framework guides our search strategy. This framework facilitates the organization of our search strategy by identifying the relevant elements of the research question and aligning them with corresponding search terms. The application of the PICO framework in orchestrating our search strategy underscores our commitment to a systematic and evidence-based review process. The detailed application of the PICO framework and the corresponding search terms and strategies is outlined in Table 1. This structured and strategic approach to literature search is pivotal in ensuring the exhaustive coverage of relevant studies, thereby contributing to the rigor and comprehensiveness of our systematic review.

Table 1. Search strategy using the PICO (Population, Intervention, Comparison, and Outcomes) framework.

PICO elements	Keywords	Search terms	Search strategies
Population	Nurses in health care settings	Nurse	(nurse*) AND
Intervention	Psychoeducational burnout intervention	Burnout intervention	((psychoeducation) OR (coping) OR (burnout intervention*) OR (cognitive behavioral therapy) OR (cognitive behavioural therapy) OR (mindfulness) OR (stress reduction)) AND
Comparison	—[a]	—	—
Outcomes	Reduce burnout	Burnout	(burnout)

[a]Not applicable.

Eligibility Criteria

Table 2 outlines the eligibility criteria based on the PICOS (Population, Intervention, Comparison, Outcomes, and Study design) model [40]. The focus will be on nurses working in health care settings worldwide. "Nurse" encompasses all licensed nursing professionals, considering international variations in terminology while "health care setting" refers to institutions where nursing care is administered. This approach embraces countries with diverse income levels, ensuring inclusivity across low-, middle-, and high-income economies. By incorporating studies from varied health care systems and contexts, we hope to obtain a comprehensive understanding of the issue at hand, accounting for differences in nursing practices, resources, and cultural factors impacting burnout and its interventions.

In addition, the review will also analyze delivery modalities, evaluation tools, and implementation challenges of burnout interventions among nurses. It aims to assess the impact of interventions on reducing burnout and address implementation hurdles, such as limited resources or resistance to change. Identifying these challenges provides insights for future interventions.

The search will only include studies with quantitative research methods, allowing for objective measurement and statistical analysis of burnout levels, hence ensuring that findings are based on measurable outcomes rather than subjective interpretations [41]. Quantitative research provides consistency and replicability through standardized tools [41], enhancing the generalizability of results to a broader population of nurses. The search will prioritize studies reporting baseline and postintervention burnout changes, allowing for a thorough evaluation of intervention effectiveness over time. Burnout assessment will form the main outcome. Validated self-reported questionnaires must be applied in the studies to ensure reliability and validity in measuring burnout, enabling standardized and objective data collection across studies. Studies within the past decade will be targeted to ensure that recent publications with updated research findings are retrieved. The review will exclude gray literature as the preliminary scan by the researchers revealed a sufficiently large number of studies with good quality and reliability and have undergone a rigorous peer-review process. The decision to exclude gray literature was made because of limited accessibility, inconsistent indexing, and difficulties in assessing the quality and reliability of information [42].

XSL•FO
RenderX

Table 2. Eligibility criteria based on the PICOS (Population, Intervention, Comparison, Outcomes, and Study design) model.

PICOS model categories	Description
Population	• Nurses working in health care settings across any country
Intervention	• All studies on interventions focused on person-directed psychoeducational approaches to address burnout among nurses in health care settings
Comparison	• An inactive control group that did not receive an intervention or received usual care, OR<br>• An active control group that received an alternative intervention for burnout
Outcomes	The following elements will be examined:<br>• Characteristics of burnout interventions<br>• Changes in burnout levels from preintervention to postintervention, including evaluation tools<br>• Implementation challenges of burnout interventions
Study design	• Publications that are written in the English language with full text and are peer-reviewed journal papers<br>• Studies conducted between January 1, 2014, and December 31, 2023<br>• All quantitative studies that involve case and control groups (randomized controlled trials, nonrandomized experimental studies, and cohort studies)

Data Management

Study Selection

Our systematic review protocol will use a meticulous multilevel screening approach to streamline the process of identifying relevant literature on psychoeducational burnout interventions for nurses. In the initial stage, study titles and abstracts will be screened, followed by a thorough examination of the full texts of the selected studies to determine the eligibility criteria. Searches, eligibility assessments, and data extraction will be performed independently in an unblinded standardized manner by all team members working in pairs. Any discrepancies will be resolved by seeking the consensus of a third team member. Next, the full-text appraisal will be performed on selected articles before the list of included studies is finalized. Similarly, any discrepancies between each pair of reviewers will be resolved through broader team discussions and mutual agreement. Subsequently, all selected studies will subsequently be imported into Google Sheets, a web-based spreadsheet editor, to facilitate organization and further review. This collaborative approach ensures a comprehensive and impartial selection of studies. Outcomes from both screening levels will be meticulously documented, adhering to the PRISMA guidelines. The screening process will be recorded through the PRISMA flow diagram in Figure 1, including the reasons for study exclusion at each stage, ensuring transparency and accountability of the study selection process. This systematic and collaborative approach to study selection and data management is crucial to ensure the integrity and validity of our systematic review.

XSL•FO
RenderX

Figure 1. Preferred Reporting Items for Systematic Review and Meta-Analysis flow diagram.

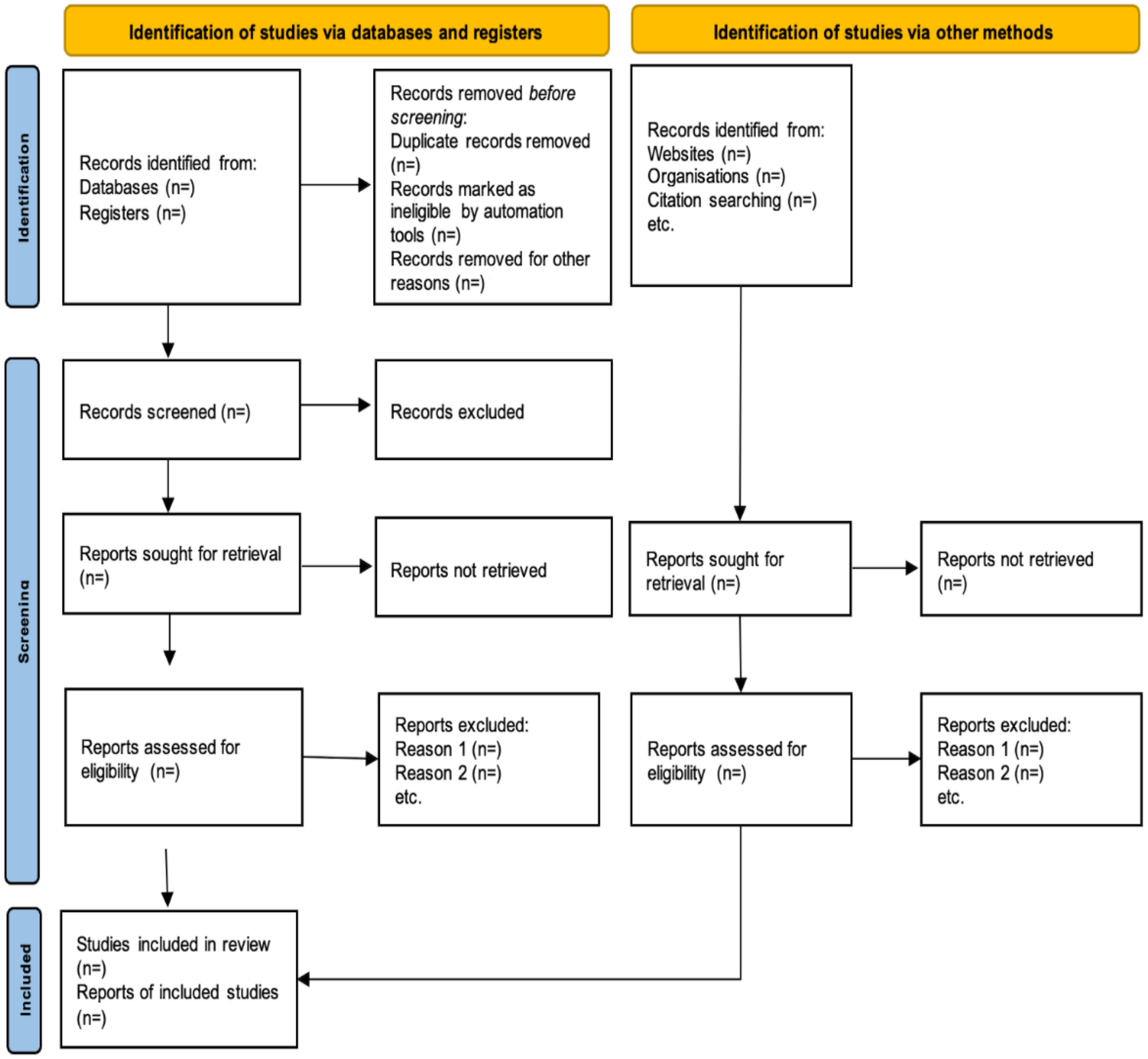

Data Extraction

Data extraction will be conducted independently and in an unblinded, standardized manner by all team members working in pairs. This approach ensures a thorough and accurate data extraction from the finalized paper. This process will use a standardized data extraction form to capture comprehensive information pertinent to our research question. Any reviewer disagreement regarding the extracted data will be resolved through discussion and consensus among all team members. To further validate any unclear data, the corresponding authors of the studies may be contacted to provide any missing information or additional details. This step is crucial to generate a more comprehensive understanding of the interventions and enhance the overall quality of the systematic review. The extracted data will encompass various aspects relevant to the current review (Table 3).

All extracted data will be documented in Google Sheets to enable efficient collaboration between all team members in organizing and analyzing the data. This approach ensures that all team members have real-time access to the data, facilitating a transparent and efficient review process. No external software or tool will be used for data extraction and management, underscoring our commitment to a straightforward and accessible method of handling the data gathered in the review. This structured approach to data extraction is designed to ensure the reliability, accuracy, and comprehensiveness of our review findings.

XSL•FO
RenderX

Table 3. Data extraction template.

Sections	Description
**Section 1: Bibliometric details**	
Authors name	For example, Smith J.
Year of publication	For example, YYYY
Title	Title of the study
Journal	Name of the journal
Country	For example, Africa
**Section 2: Study characteristics**	
Study design	Provide the study design used in the study
Study location	Provide the location where the study was conducted
Sample size	Provide the total number of participants in the intervention and control group
Target population	Provide the target population in the study
**Section 3: Intervention characteristics**	
Name of intervention	Provide the name of the intervention
Content of intervention	Describe the content of the intervention
Implementer	Provide the facilitator of the intervention
Frequency of intervention	Provide frequency of implemented intervention to participants of the study
Duration of intervention implemented	Provide duration from the start of intervention until the end
Comparison control group	Provide information if the comparison group was given any intervention
Burnout measurement tool	Provide the name of the burnout measurement tool used in the study
Follow-up frequency	Provide a timeline of when pre- and postintervention follow-up is done
**Section 4: Outcome**	
Outcome of intervention	Describe reported burnout level pre- and post-intervention
Facilitator factors	Provide facilitators identified, if available
Barrier factors	Provide barriers identified, if available
Limitation of study	Provide limitations of study, if available

Quality Appraisal

The methodological quality of the studies included in our review will be rigorously assessed using the critical appraisal tools developed by the Joanna Briggs Institute (JBI), an esteemed international research organization that has developed various tools for evaluating the effectiveness and appropriateness of health care interventions. Specifically, the JBI Critical Appraisal Tool for Assessment of Risk of Bias for Randomized Controlled Trials [43] and JBI Critical Appraisal Checklist for Quasi-Experimental Studies [44], which feature 13 and 9 questions respectively, will be used to evaluate the reporting quality of the papers. These tools will facilitate a detailed examination of internal validity aspects, including biases related to selection and allocation, administration of intervention or exposure, assessment, detection and measurement of outcomes, participant retention, as well as statistical conclusion validity. The responses to the questions are categorized as "yes," "no," "unclear," and "not applicable." A study is considered "unclear" if it does not provide explicit information for a specific question. If a question is irrelevant to the study's context, it is labeled as "not applicable." A scoring system will be applied, where when a question is answered with "yes," it is assigned a score of 1 point. However, if the answer is "no," "unclear," or "not applicable" no points are awarded. Based on these responses, the quality scores will be organized and displayed in a table format. To ensure a comprehensive and objective evaluation, 2 reviewers will evaluate the quality of each study independently, with their ratings remaining confidential to each other to maintain impartiality. Any discrepancies in the assessment of the studies will be addressed through discussion among the reviewers until a consensus is reached. A third reviewer will be consulted to provide additional input and facilitate resolution if necessary. In addition to assessing the risk of bias in individual studies, the overall quality of evidence across all the papers will also be evaluated. This meticulous quality appraisal process aims to provide a robust understanding of the methodological strengths and limitations of the included studies, bolstering confidence in the review findings.

Data Synthesis

In the data synthesis phase, all the included studies will be examined comprehensively. Study characteristics and quality, as well as the effects of the interventions on nurse burnout, will

XSL•FO
RenderX

be tabulated. This process will be underpinned by a narrative synthesis approach to organize and summarize and organize the extracted information from each study thematically. This approach enables the identification of effective psychoeducational interventions for managing nurses' burnout levels.

An integral part of our data synthesis will involve conducting subgroup analyses to assess the differential effectiveness of psychoeducational programs among nurses. This analysis is designed to discern specific characteristics that may influence the outcomes of psychoeducational interventions. We will categorize subgroups based on the type of psychoeducational intervention received, including online programs, in-person workshops, and blended learning formats. Through this meticulous approach, we aim to determine how the mode of delivery impacts intervention effectiveness, thus contributing valuable insights into optimizing burnout management strategies within the nursing profession.

To ensure transparency and adherence to reporting standards, the PRISMA guidelines will be followed when reporting the results. These guidelines provide a structured framework for reporting systematic reviews and meta-analyses, ensuring the findings are presented comprehensively and standardized. By adhering to these guidelines, the review will maintain a high level of methodological rigor and enhance the credibility and reproducibility of the results.

Results

The study was initiated in February 2024 by forming a research team and assigning respective roles. The systematic literature review was commenced in February 2024, and data synthesis was expected to be concluded by May 2024 and the review by June 2024. The findings will be used in developing a psychoeducation program tailored for nurses in our country. Further dissemination to an international audience will also be undertaken through peer-reviewed journal publications and scientific presentations (PRISMA flowchart provided in Figure 1).

Discussion

Expected Results and Practical Implications

This systematic review is important because it comprehensively maps existing research and literature pertaining to psychoeducation as a tool for managing burnout among nurses. By systematically analyzing a wide range of intervention strategies across diverse health care settings, this review will explore the most effective methods and contribute to a deeper understanding of burnout intervention, providing valuable insights for health care practitioners and policymakers. This systematic review marks the initial phase of a comprehensive research effort to develop a person-directed psychoeducational burnout intervention package tailored for nurses within our country. The primary objective of this review is to identify and evaluate person-directed psychoeducational interventions that have demonstrated effectiveness in mitigating burnout among nurses. This review will serve as a foundational framework for developing comprehensive intervention packages tailored to nurses' specific needs in various health care settings. By incorporating diverse methodologies, durations, and delivery formats, the review aims to offer a holistic view of effective strategies.

To the best of our knowledge, no other work has systematically reviewed the effectiveness of psychoeducational intervention in mitigating burnout among nurses. This review is anticipated to provide multi-faceted outcomes that contribute to research and practice in nursing and health management. As a start, we will identify effective psychoeducation-based person-directed intervention strategies tailored to address burnout among nurses that can be incorporated as pivotal components of the intervention package to reduce burnout and promote well-being among nurses. Highlighting the challenges and successes inherent in implementing these interventions will offer detailed insights into their practical implementation alongside real-world applicability and scalability considerations. Furthermore, this review places great focus on the practical implications of applying these interventions in real-world settings. Understanding the challenges and accomplishments of these interventions will provide valuable insights into their feasibility, scalability, and applicability. This knowledge is critical for health care organizations that want to incorporate evidence-based methods into their daily operations to help nurses' mental health and well-being.

Empowering nurses and other HCPs with the knowledge and resources is a prerequisite for proactive burnout mitigation. Thus, the insights garnered from this review will inform the development of evidence-based interventions and policies for seamlessly integrating psychoeducation and person-directed approaches into existing practices that prevent and manage burnout among nurses. This review may stimulate improvements in job satisfaction, retention rates, and overall patient care quality. Furthermore, effective burnout interventions hold the potential to yield substantial cost savings for health care systems by mitigating turnover rates, absenteeism, and medical errors associated with burnout, thereby enhancing the standard and safety of patient care delivery. Empowering nurses with knowledge and resources for proactive burnout mitigation is essential. The findings from this review will inform the development of evidence-based interventions and policies, integrating psychoeducation and person-directed approaches to prevent and manage burnout among nurses. These insights could lead to improved job satisfaction, retention rates, and overall patient care quality. Effective burnout interventions also hold the potential to yield substantial cost savings for health care systems by reducing turnover rates, absenteeism, and medical errors associated with burnout.

Last but not least, this review will identify notable gaps in this literature and areas and guide future research endeavors. In-depth investigations into the efficacy and implementation of psychoeducation-based person-directed interventions for burnout prevention and management among nurses will foster continuous progress and innovation in the field, besides providing insight into similar adaptation for other HCPs.

XSL•FO
RenderX

Limitations

This review is subjected to certain limitations. First, since our search was conducted solely using electronic databases, the exclusion of unpublished or gray literature may potentially result in the oversight of some studies. The omission of gray literature might lead to losing valuable insights or unpublished findings regarding burnout interventions among nurses. However, including gray literature may complicate systematic retrieval and quality assessment, potentially affecting overall reliability and introducing heterogeneity.

Second, our decision to restrict the review to English-language publications could narrow its scope by possibly excluding relevant studies in other languages. We opted for English-language studies due to practical considerations, that is the broader accessibility of English publications and limited translation resources. In addition, our focus on studies from the past decade was intended to capture this landscape of burnout interventions, but this approach may introduce selection bias.

Conclusion

Given the increasing prevalence of burnout among nurses and its adverse effects on both individuals and organizations, this systematic review is timely and crucial. The findings are expected to provide a robust evidence base for health care policy makers and practitioners to develop and implement effective psychoeducational interventions tailored to the specific needs of nurses. By identifying successful strategies and understanding the barriers to their implementation, this review aims to support the development of targeted approaches to manage nurse burnout. In the long run, enhancing nurses' well-being will lead to improved patient care quality and overall health care delivery, benefiting both health care providers and recipients.

Acknowledgments

The authors thank the Director General of Health Malaysia for granting permission to publish this protocol. In addition, we would like to thank the Sector for evidence-based health care, the National Institutes of Health, Malaysia, for their guidance in conducting this review. This research received no specific grant from any funding agency in the public, commercial, or not-for-profit sectors.

Data Availability

All data generated and analyzed in this study are available upon request from the corresponding author.

Authors' Contributions

All authors made equal contributions to this work. All authors have reviewed and approved the protocol.

Conflicts of Interest

None declared.

Multimedia Appendix 1

PRISMA-P (Preferred Reporting Items for Systematic Review and Meta-Analysis Protocols) checklist.
[DOCX File , 34 KB-Multimedia Appendix 1]

References

1. Aase K, Guise V, Billett S, Sollid SJM, Njå O, Røise O, et al. Resilience in healthcare (RiH): a longitudinal research programme protocol. BMJ Open. 2020;10(10):e038779. [FREE Full text] [doi: 10.1136/bmjopen-2020-038779] [Medline: 33109657]
2. Sener T, Haenen W, Smits P, Hans GH. Large-scale real-life implementation of technology-enabled care to maximize hospitals' medical surge preparedness during future infectious disease outbreaks and winter seasons: a viewpoint. Front Public Health. 2023;11:1149247. [FREE Full text] [doi: 10.3389/fpubh.2023.1149247] [Medline: 37621607]
3. Sato C, Adumattah A, Abulencia MK, Garcellano PD, Li ATW, Fung K, et al. COVID-19 mental health stressors of health care providers in the pandemic acceptance and commitment to empowerment response (PACER) intervention: qualitative study. JMIR Form Res. 2022;6(3):e35280. [FREE Full text] [doi: 10.2196/35280] [Medline: 35138256]
4. Alsaeed D, Al-Ozairi A, Alsarraf H, Albarrak F, Al-Ozairi E. Are we ready for the next pandemic? Lessons learned from healthcare professionals' perspectives during the COVID-19 pandemic. Front Public Health. 2023;11:1048283. [FREE Full text] [doi: 10.3389/fpubh.2023.1048283] [Medline: 37064685]
5. Maslach C, Schaufeli WB, Leiter MP. Job burnout. Annu Rev Psychol. 2001;52(1):397-422. [doi: 10.1146/annurev.psych.52.1.397] [Medline: 11148311]
6. Brady KJS, Ni P, Sheldrick RC, Trockel MT, Shanafelt TD, Rowe SG, et al. Describing the emotional exhaustion, depersonalization, and low personal accomplishment symptoms associated with maslach burnout inventory subscale scores in US physicians: an item response theory analysis. J Patient Rep Outcomes. 2020;4(1):42. [FREE Full text] [doi: 10.1186/s41687-020-00204-x] [Medline: 32488344]

XSL•FO
RenderX

7. Mark G, Smith AP. Occupational stress, job characteristics, coping, and the mental health of nurses. Br J Health Psychol. 2012;17(3):505-521. [doi: 10.1111/j.2044-8287.2011.02051.x] [Medline: 22107162]
8. Dall'Ora C, Ball J, Reinius M, Griffiths P. Burnout in nursing: a theoretical review. Hum Resour Health. 2020;18(1):41. [FREE Full text] [doi: 10.1186/s12960-020-00469-9] [Medline: 32503559]
9. Ramli SN, Ahayalimudin N. Burnout level and its associated factors among critical care nurses: a literature review. Int J Care Scholars. 2023;6(1):59-70. [doi: 10.31436/ijcs.v6i1.277]
10. Scerri J, Sammut A, Mangion M, Galea M, Grech P, Cilia Vincenti S, et al. Targeting burnout and emotional state in nurses within a mental health care setting: a quasi-experimental study. HSSR. 2022;6(1):p1. [doi: 10.30560/hssr.v6n1p1]
11. Mudallal RH, Othman WM, Al Hassan NF. Nurses' burnout: the influence of leader empowering behaviors, work conditions, and demographic traits. Inq J Health Care Organ Provis Financ. 2017;54:46958017724944. [FREE Full text] [doi: 10.1177/0046958017724944] [Medline: 28844166]
12. Heinen MM, van Achterberg T, Schwendimann R, Zander B, Matthews A, Kózka M, et al. Nurses' intention to leave their profession: a cross sectional observational study in 10 European countries. Int J Nurs Stud. 2013;50(2):174-184. [doi: 10.1016/j.ijnurstu.2012.09.019] [Medline: 23107005]
13. Woo T, Ho R, Tang A, Tam W. Global prevalence of burnout symptoms among nurses: a systematic review and meta-analysis. J Psychiatr Res. 2020;123:9-20. [doi: 10.1016/j.jpsychires.2019.12.015] [Medline: 32007680]
14. Abd Wahab AT, Mohd Hairon S, Shafei MN, Ibrahim MI, Mahmud N. Exploring job stress among public health workforce in northeastern Malaysia. Cureus. 2023;15(11):e49083. [FREE Full text] [doi: 10.7759/cureus.49083] [Medline: 38125210]
15. Zakaria N, Zakaria NH, Bin Abdul Rassip MNA, Lee KY. Burnout and coping strategies among nurses in Malaysia: a national-level cross-sectional study. BMJ Open. 2022;12(10):e064687. [FREE Full text] [doi: 10.1136/bmjopen-2022-064687] [Medline: 36216421]
16. Salvagioni DAJ, Melanda FN, Mesas AE, González AD, Gabani FL, Andrade SMD. Physical, psychological and occupational consequences of job burnout: a systematic review of prospective studies. PLoS One. 2017;12(10):e0185781. [FREE Full text] [doi: 10.1371/journal.pone.0185781] [Medline: 28977041]
17. Baye Y, Demeke T, Birhan N, Semahegn A, Birhanu S. Nurses' work-related stress and associated factors in governmental hospitals in Harar, Eastern Ethiopia: a cross-sectional study. PLoS One. 2020;15(8):e0236782. [FREE Full text] [doi: 10.1371/journal.pone.0236782] [Medline: 32745142]
18. Jun J, Ojemeni MM, Kalamani R, Tong J, Crecelius ML. Relationship between nurse burnout, patient and organizational outcomes: systematic review. Int J Nurs Stud. 2021;119:103933. [doi: 10.1016/j.ijnurstu.2021.103933] [Medline: 33901940]
19. Kath LM, Stichler JF, Ehrhart MG, Sievers A. Predictors of nurse manager stress: a dominance analysis of potential work environment stressors. Int J Nurs Stud. 2013;50(11):1474-1480. [doi: 10.1016/j.ijnurstu.2013.02.011] [Medline: 23522937]
20. Tamata AT, Mohammadnezhad M. A systematic review study on the factors affecting shortage of nursing workforce in the hospitals. Nurs Open. 2023;10(3):1247-1257. [FREE Full text] [doi: 10.1002/nop2.1434] [Medline: 36303066]
21. Czeglédi E, Tandari-Kovács M. [Characteristics and prevention of burnout syndrome among nurses]. Orv Hetil. 2019;160(1):12-19. [doi: 10.1556/650.2019.30856] [Medline: 30599782]
22. Muir KJ, Wanchek TN, Lobo JM, Keim-Malpass J. Evaluating the costs of nurse burnout-attributed turnover: a markov modeling approach. J Patient Saf. 2022;18(4):351-357. [doi: 10.1097/PTS.0000000000000920] [Medline: 35617593]
23. Awa WL, Plaumann M, Walter U. Burnout prevention: a review of intervention programs. Patient Educ Couns. 2010;78(2):184-190. [doi: 10.1016/j.pec.2009.04.008] [Medline: 19467822]
24. Cohen C, Pignata S, Bezak E, Tie M, Childs J. Workplace interventions to improve well-being and reduce burnout for nurses, physicians and allied healthcare professionals: a systematic review. BMJ Open. 2023;13(6):e071203. [FREE Full text] [doi: 10.1136/bmjopen-2022-071203] [Medline: 37385740]
25. DeChant PF, Acs A, Rhee KB, Boulanger TS, Snowdon JL, Tutty MA, et al. Effect of organization-directed workplace interventions on physician burnout: a systematic review. Mayo Clin Proc Innov Qual Outcomes. 2019;3(4):384-408. [FREE Full text] [doi: 10.1016/j.mayocpiqo.2019.07.006] [Medline: 31993558]
26. Irina GC, Isabelle B, Yara S. O-192 Effectiveness of organizational interventions to prevent occupational burnout: a systematic review and meta-analysis. BMJ. 2023;80(1):A66. [doi: 10.1136/oem-2023-epicoh.161]
27. Tamminga SJ, Emal LM, Boschman JS, Levasseur A, Thota A, Ruotsalainen JH, et al. Individual-level interventions for reducing occupational stress in healthcare workers. Cochrane Database Syst Rev. 2023;5(5):CD002892. [FREE Full text] [doi: 10.1002/14651858.CD002892.pub6] [Medline: 37169364]
28. Ruotsalainen J, Serra C, Marine A, Verbeek J. Systematic review of interventions for reducing occupational stress in health care workers. Scand J Work Environ Health. 2008;34(3):169-178. [FREE Full text] [doi: 10.5271/sjweh.1240] [Medline: 18728906]
29. Selič-Zupančič P, Klemenc-Ketiš Z, Onuk Tement S. The impact of psychological interventions with elements of mindfulness on burnout and well-being in healthcare professionals: a systematic review. J Multidiscip Healthc. 2023;16:1821-1831. [FREE Full text] [doi: 10.2147/JMDH.S398552] [Medline: 37404957]
30. Nielsen K, Christensen M. Positive participatory organizational interventions: a multilevel approach for creating healthy workplaces. Front Psychol. 2021;12:696245. [FREE Full text] [doi: 10.3389/fpsyg.2021.696245] [Medline: 34262513]

XSL•FO
RenderX

31. Odole AC, Ogunlana MO, Odunaiya NA, Oyewole OO, Mbada CE, Onyeso OK, et al. Influence of well-being and quality of work-life on quality of care among healthcare professionals in southwest, Nigeria. Sci Rep. 2023;13(1):7830. [FREE Full text] [doi: 10.1038/s41598-022-25057-w] [Medline: 37188741]
32. Zhang XJ, Song Y, Jiang T, Ding N, Shi TY. Interventions to reduce burnout of physicians and nurses: an overview of systematic reviews and meta-analyses. Medicine (Baltimore). 2020;99(26):e20992. [FREE Full text] [doi: 10.1097/MD.0000000000020992] [Medline: 32590814]
33. Lee M, Cha C. Interventions to reduce burnout among clinical nurses: systematic review and meta-analysis. Sci Rep. 2023;13(1):10971. [FREE Full text] [doi: 10.1038/s41598-023-38169-8] [Medline: 37414811]
34. Suleiman-Martos N, Gomez-Urquiza JL, Aguayo-Estremera R, Cañadas-De La Fuente GA, De La Fuente-Solana EI, Albendín-García L. The effect of mindfulness training on burnout syndrome in nursing: a systematic review and meta-analysis. J Adv Nurs. 2020;76(5):1124-1140. [doi: 10.1111/jan.14318] [Medline: 32026484]
35. Sulosaari V, Unal E, Cinar FI. The effectiveness of mindfulness-based interventions on the psychological well-being of nurses: a systematic review. Appl Nurs Res. 2022;64:151565. [FREE Full text] [doi: 10.1016/j.apnr.2022.151565] [Medline: 35307128]
36. Hsu HC, Lee HF, Hung HM, Chen YL, Yen M, Chiang HY, et al. Effectiveness of individual-based strategies to reduce nurse burnout: an umbrella review. Journal of Nursing Management. 2024;2024:1-13. [doi: 10.1155/2024/8544725]
37. Page MJ, Moher D, Bossuyt PM, Boutron I, Hoffmann TC, Mulrow CD, et al. PRISMA 2020 explanation and elaboration: updated guidance and exemplars for reporting systematic reviews. BMJ. 2021;372:n160. [FREE Full text] [doi: 10.1136/bmj.n160] [Medline: 33781993]
38. Sharin IA, Jinah N, Bakit P. Psychoeducational burnout intervention for nurses: a systematic review. PROSPERO. 2024. URL: https://www.crd.york.ac.uk/prospero/display_record.php?ID=CRD42024505762 [accessed 2024-08-31]
39. Page MJ, McKenzie JE, Bossuyt PM, Boutron I, Hoffmann TC, Mulrow CD, et al. The PRISMA 2020 statement: an updated guideline for reporting systematic reviews. BMJ. 2021;372:n71. [FREE Full text] [doi: 10.1136/bmj.n71] [Medline: 33782057]
40. Methley AM, Campbell S, Chew-Graham C, McNally R, Cheraghi-Sohi S. PICO, PICOS and SPIDER: a comparison study of specificity and sensitivity in three search tools for qualitative systematic reviews. BMC Health Serv Res. 2014;14:579. [FREE Full text] [doi: 10.1186/s12913-014-0579-0] [Medline: 25413154]
41. Hagan TL. Measurements in quantitative research: how to select and report on research instruments. Oncol Nurs Forum. 2014;41(4):431-433. [doi: 10.1188/14.ONF.431-433] [Medline: 24969252]
42. Gul S, Shah TA, Ahmad S, Gulzar F, Shabir T. Is grey literature really grey or a hidden glory to showcase the sleeping beauty. Collect Curation. 2020;40(3):100-111. [doi: 10.1108/cc-10-2019-0036]
43. Barker TH, Stone JC, Sears K, Klugar M, Tufanaru C, Leonardi-Bee J, et al. The revised JBI critical appraisal tool for the assessment of risk of bias for randomized controlled trials. JBI Evid Synth. 2023;21(3):494-506. [doi: 10.11124/JBIES-22-00430] [Medline: 36727247]
44. Barker TH, Habibi N, Aromataris E, Stone JC, Leonardi-Bee J, Sears K, et al. The revised JBI critical appraisal tool for the assessment of risk of bias for quasi-experimental studies. JBI Evid Synth. 2024;22(3):378-388. [doi: 10.11124/JBIES-23-00268] [Medline: 38287725]

Abbreviations

HCP: Health Care Professional
***ICD-11*:** *International Classification of Diseases, 11th Revision*
JBI: Joanna Briggs Institute
PICO: Population, Intervention, Comparison, and Outcomes
PICOS: Population, Intervention, Comparison, Outcomes, and Study design
PRISMA: Preferred Reporting Items for Systematic Review and Meta-Analysis
PROSPERO: International Prospective Register of Systematic Reviews

Edited by T Leung, S Ma; submitted 27.03.24; peer-reviewed by D Whybrow; comments to author 29.05.24; revised version received 18.06.24; accepted 16.07.24; published 30.09.24

Please cite as:
Abdullah Sharin I, Jinah N, Bakit P, Adnan IK, Zakaria NH, Mohmad S, Ahmad Subki SZ, Zakaria N, Lee KY
Psychoeducational Burnout Intervention for Nurses: Protocol for a Systematic Review
JMIR Res Protoc 2024;13:e58692
URL: https://www.researchprotocols.org/2024/1/e58692
doi: 10.2196/58692
PMID:

XSL•FO
RenderX

©Ili Abdullah Sharin, Norehan Jinah, Pangie Bakit, Izzuan Khirman Adnan, Nor Haniza Zakaria, Shazwani Mohmad, Siti Zubaidah Ahmad Subki, Nursyahda Zakaria, Kun Yun Lee. Originally published in JMIR Research Protocols (https://www.researchprotocols.org), 30.09.2024. This is an open-access article distributed under the terms of the Creative Commons Attribution License (https://creativecommons.org/licenses/by/4.0/), which permits unrestricted use, distribution, and reproduction in any medium, provided the original work, first published in JMIR Research Protocols, is properly cited. The complete bibliographic information, a link to the original publication on https://www.researchprotocols.org, as well as this copyright and license information must be included.

XSL•FO
RenderX
